# Supplementary material for: A retrofit sensing strategy for soft fluidic robots
Source: Nat Commun. 2024 Jan 15;15:539. doi: 10.1038/s41467-023-44517-z (PMC10789869; doi:10.1038/s41467-023-44517-z)
Supplement: Supplementary file 1 — Supplementary Information [file 41467_2023_44517_MOESM1_ESM.pdf]

Supplementary information for

A Retrofit Sensing Strategy for Soft Fluidic Robots

Shibo Zou[1], Sergio Picella[1,2], Jelle de Vries[1], Vera G. Kortman[3,4], Aimée Sakes[4], Johannes T. B. Overvelde[1,2]

[1] Autonomous Matter Department, AMOLF, Amsterdam, 1098 XG, The Netherlands

[2] Institute for Complex Molecular Systems and Department of Mechanical Engineering, Eindhoven University of Technology, Eindhoven, 5600 MB, The Netherlands

[3] Department of Marine and Transport Technology, Delft University of Technology, Delft, 2628 CD, The Netherlands

[4] Bio-Inspired Technology Group, Department of BioMechanical Engineering, Delft University of Technology, Delft, 2628 CD, The Netherlands

Corresponding author: Johannes T. B. Overvelde, overvelde@amolf.nl

This document includes:

Methods sections

Supplementary references

Supplementary tables S1-S3

Supplementary figures S1-S35

Methods

Fabrication of PneuNet actuators and soft gripper

The PneuNet actuator was molded in a two-step process (Fig. S22). In the first step, the extensible layer that consists of air chambers was molded with Dragon Skin (DS) 10 Medium silicone (Smooth-On). Since the DS silicone cures in 5 hours, we waited 3.5 hours for the DS silicone to be almost cured. In the second step, we molded the Elite Double (ED) 32 silicone (Zhermack) around the almost-cured DS silicone to form the inextensible layer and the inlet unit. This way, the ED silicone could cure together with the DS silicone, leading to a strong adhesion between the extensible and inextensible layers. A grid fabric (Penelope 70/10, Garenenzo) was embedded during the molding to further increase the axial stiffness of the inextensible layer. We used one sacrificial inner mold and one reconfigurable outer mold containing six parts for the two-step molding process. The inner mold was printed with butene-diol vinyl alcohol (BVOH) on a Fused Filament Fabrication (FFF) 3D printer (Ultimaker 3). The outer mold parts were printed with an acrylic-like photopolymer (VeroClear, Stratasys) on a PolyJet 3D printer (Eden260VS, Stratasys). The VeroClear mold tends to inhibit the curing of silicone, especially those freshly printed. Therefore, we brushed a thin layer of anti cure inhibition coating (Inhibit X, Smooth-On) onto the inner surface of the VeroClear mold to avoid the cure inhibition. The outer molds can be assembled into two different configurations corresponding to the two steps of the molding process. In each step, the silicone was first loaded into a two-component cartridge (AF 400-01-10-01, Sulzer), degassed and then injected into the mold through a static mixing nozzle (MFQ 05-24L) using a pneumatic extrusion gun. Before the molding of each silicone, a thin layer of release agent (Ease release 200, Smooth-On) was sprayed and then brushed evenly over the molding surfaces. The silicones were cured at room temperature. After curing, the BVOH inner mold was dissolved and flushed out by connecting the actuator to a water pump

(H5750010, FLOJET) in parallel with a tunable flow resistor for venting. The pneumatic connectors for the single PneuNet actuator and the soft grippper were printed with VeroClear on the Stratasys 3D printer. Vacuum grease was brushed into the connector, and the PneuNet actuator was gently pressed into the connector.

Distance sensing with PneuNet bending actuator

The pneumatic connector containing a single PneuNet actuator was mounted on an aluminium frame to ensure that the bottom edge of the actuator at rest stays horizontal under gravity. The actuator was inflated onto a horizontal aluminium bar from a distance h. We adjusted h from 0 mm to 40 mm in 5 mm increments by manually moving the aluminium bar with a caliper. To control and measure the fluidic tests, we used a data acquisition card (NI-DAQ USB-6212, National Instruments) with custom software developed at AMOLF. The measurement setup contains (depending on the sensing method used) a proportional pressure regulator (-100 kPa to 100 kPa, VEAB-L-26-D13-Q4-V1-1R1, Festo), mass flow controller (SLA5850, Brooks Instrument), 3/2-way solenoid valve (VDW250-5G-2-01F-Q, SMC), air tanks (CRVZS-0.1, CRVZS-0.75, Festo), bidirectional flow sensor (HAFBLF0750CAAX5, Honeywell), $\pm$ 34.5 kPa and $\pm$ 103.4 kPa pressure sensors (SSCDRRN005PDAA5, SSCDRRN015PDAA5, Honeywell). All measurements were done with a data acquisition frequency of 1000 Hz. All the tests conducted with the data acquisition card in this paper started with 15 s blank measurement for sensor offset, and the data after offset were smoothed in MATLAB using the loess method (local regression using weighted linear least squares and a second degree polynomial model) with a span of 20 data points to remove high frequency noise before any further analysis.

To obtain the pressure-volume curves in Fig. 1a, we measured both the flow input and pressure response over time as the actuator was inflated onto the aluminium bar from a distance h. The actuation system (Fig. S23) included, in the direction of air flow, a mass flow controller and

a 3/2-way solenoid valve. The input port of the mass flow controller was connected to the compressed air source that was set at 150 kPa by an independent wall-mounted pressure regulator (LRP-1/4-2.5, Festo). We added a 0.75 L air tank as a buffer in between the wall-mounted pressure regulator and mass flow controller. The normally closed port of the valve was connected to the mass flow controller, the normally open port of the valve was connected to the atmosphere, and the inlet port of the valve was connected to the PneuNet actuator through a bidirectional flow sensor. We added a flow resistor made from Teflon tube and nozzles (920050-TE, Metcal) with an equivalent flow resistance around $1.8 \times 10^9\ \mathrm{Pa} \cdot \mathrm{s/m^3}$ in between the valve and flow sensor to ensure that the flow rate was within the sensor range. We used a $\pm$ 103.4 kPa pressure sensor to measure the pressure in the actuator. A total of 8 actuation cycles were performed at each h by programming the custom software for the data acquisition system. Each actuation cycle started by switching the valve on, increasing the flow rate from 0 to 0.6 SLPM (standard liter per minute) in 5 s, holding the flow rate at 0.6 SLPM for 2 s, then decreasing the flow rate to 0 SLPM, holding at 0 SLPM for 30 s, and finally switching the valve off and discharging the actuator for 30 s. For each h, the pressure-volume curve was fitted from the measurements of the last 5 actuation cycles using a six-order polynomial with a fixed zero y-intercept.

We come up with four practical methods to extract the sensing information and demonstrate here by distance sensing with a soft PneuNet bending actuator: 1) pressure control with pressure measurement; 2) pressure control with flow measurement; 3) flow control with pressure measurement; 4) pressure-time measurement (Time of first contact in Section Time-enabled sensing versatility). Methods 1) to 3) measure the fluidic signal at the equilibrium state, which is related with the final shape of the soft actuator after the interaction with the environment, while method 4) can give us more information about when and how many contact events happened. To implement this sensing strategy in practice, each method requires different hardware which may scale up with the number of fluidic sources. Method 1) requires an air tank and a valve for

each fluidic source and a pressure sensor for each actuator. Method 2) and 3) requires no additional devices for each fluidic source but a flow sensor and pressure sensor for each actuator, respectively. Method 4) can be done with any of the systems above.

Pressure control with pressure measurement

The actuation system (Fig. S24) included, in the direction of air flow, a proportional pressure regulator, a 3/2-way solenoid valve, a 0.1 L air tank, and another 3/2-way solenoid valve. The input port of the proportional pressure regulator was connected to the compressed air source that was set at 150 kPa by an independent wall-mounted pressure regulator. For the solenoid valve, the inlet port was connected to the proportional pressure regulator and the normally closed port was connected to the air tank, the normally open port was blocked by a cap (FTLLP-6005, Nordson). For the second valve, the inlet port was connected to the PneuNet actuator and the normally closed port was connected to the air tank, the normally open port was connected to the atmosphere. We used a $\pm$ 103.4 kPa pressure sensor and a $\pm$ 34.5 kPa pressure sensor to measure the pressure in the 0.1 L air tank and actuator, respectively. A flow resistor with an equivalent flow resistance around $7.3 \times 10^9 \ \mathrm{Pa \cdot s/m^3}$ was added between the second solenoid valve and the actuator to avoid any dynamic effect during the charging of the actuator. A total of 8 actuation cycles were performed at each h by programming the custom software for the data acquisition system. Each actuation cycle started by setting the proportional pressure regulator at 49.9 kPa and waiting for 10 s, then switching on the first valve to charge the 0.1 L air tank for 10 s, switching off the first valve and waiting for 10 s, then switching on the second valve to charge the actuator for 60 s, and finally switching off the second valve to discharge the actuator for 30 s. To obtain the calibration curve in Fig. 1b, an average pressure was first taken for each cycle over the last 10 s during the 60 s actuator charging period, then those average pressure values from the last 7 cycles were plotted for each h in Fig. 1b.

Pressure control with flow measurement

The actuation system (Fig. S25) included, in the direction of air flow, a proportional pressure regulator and a 3/2-way solenoid valve. The input port of the proportional pressure regulator was connected to the compressed air source that was set at 100 kPa by an independent wall-mounted pressure regulator. The normally closed port of the valve was connected to the output port of the proportional pressure regulator. We added a 0.75 L air tank as a buffer in between the proportional pressure regulator and the valve. The normally open port of the valve was connected to the atmosphere, and the inlet port of the valve was connected to the PneuNet actuator through a bidirectional flow sensor. We added a flow resistor with an equivalent flow resistance around $1.8 \times 10^9~\mathrm{Pa} \cdot \mathrm{s/m^3}$ in between the valve and flow sensor to ensure that the flow rate lies within the sensor range. We used a 0-100 kPa pressure sensor (MPX5100DP, NXP) to measure the pressure in the actuator. A total of 8 actuation cycles were performed at each h by programming the custom software for the data acquisition system. Each actuation cycle started by setting the proportional pressure regulator at 23.8 kPa and waiting for 10 s, then switching the valve on and charging the actuator for 30 s, and finally switching the valve off and discharging the actuator for 30 s. To obtain the calibration curve in Fig. 1c, the total volume of the air going into the actuator during each cycle was calculated by integrating the flow rate over the 30 s charging period. The total volume values of the last 7 cycles were plotted for each h in Fig. 1c.

Flow control with pressure measurement

The actuation system (Fig. S23) was the same as the one for the characterization of pressure-volume curves mentioned above, except that we used a $\pm$ 34.5 kPa pressure sensor to measure the pressure in the actuator. A total of 8 actuation cycles were performed at each h by programming the custom software for the data acquisition system. Each actuation cycle started by

switching the valve on, increasing the flow rate from 0 to 0.3 SLPM (standard liter per minute) in 5 s, holding the flow rate at 0.3 SLPM for 4 s, then decreasing the flow rate to 0 SLPM, holding at 0 SLPM for 30 s, and finally switching the valve off and discharging the actuator for 30 s. To obtain the calibration curve in Fig. 1d, an average pressure was first taken for each cycle over the last 5 s during the 30 s holding period, then those average pressure values from the last 7 cycles were plotted for each h in Fig. 1d.

Size sensing with soft gripper

Five cylinders with the same height of 40 mm and diameters of 20 mm, 40 mm, 60 mm, 80 mm, and 100 mm were printed with polylactic acid (PLA) filament on a fused filament fabrication (FFF) 3D printer (Ultimaker 3). The pneumatic connector that contains the soft gripper was mounted on a robotic arm (Universal Robots UR5e) and moved to a position above the cylinder that ensures gripping with actuator tips upon inflation (Fig. 1e). The robotic arm stayed still during test and each cylinder was placed under the gripper manually without accurate alignment. The actuation system (Fig. S26) included, in the direction of air flow, a proportional pressure regulator (-100 kPa to 100 kPa, VEAB-L-26-D13-Q4-V1-1R1, Festo), a 3/2-way solenoid valve, three 0.1 L air tanks that were connected in series and another 3/2-way solenoid valve. The input port of the proportional pressure regulator was connected to the compressed air source that was set at 150 kPa by an independent wall-mounted pressure regulator. We added a 0.75 L air tank as a buffer in between the proportional pressure regulator and the first valve. For the first valve, the inlet port was connected to the proportional pressure regulator and the normally open port was connected to the air tank, the normally closed port was blocked by a cap (FTLLP-6005, Nordson). For the second valve, the inlet port was connected to the PneuNet actuator and the normally closed port was connected to the air tank, the normally open port was connected to the atmosphere. We used two $\pm$ 103.4 kPa pressure sensors to measure the pressure in the air

tanks and soft gripper, respectively. We added a small flow resistor in between the second valve and the gripper to avoid any dynamic effect during the actuation of the gripper. All control and measurement were done on the data acquisition card with custom software. For each cylinder, the proportional pressure regulator was set at 63 kPa at the beginning of the test. A total of 6 actuation cycles were performed for each cylinder by programming the custom software for the data acquisition system. Each actuation cycle started by switching off the first valve to charge the three 0.1 L air tanks for 10 s, then switching on the first valve to disconnect the tanks from the proportional pressure regulator and waiting for 10 s, switching on the second valve to charge the gripper for 20 s, then switching off the second valve to discharge the gripper. To obtain the calibration curve in Fig. 1f, an average pressure was first taken for each actuation cycle over the measurements between 4 s and 5 s after switching on the second valve. Then those average pressure values from the last 5 cycles were plotted for each D in Fig. 1f.

Time-enabled sensing versatility

Time of first contact

The analysis in this section was performed with the test results obtained from section Pressure Control with Pressure Measurement. An additional test of free actuation ($h = \infty$) was done using the same test setup and the actuator pressure-time curve from the last cycle out of the 8 cycles in total was used as a reference response for the following analysis. The actuator tip vertical displacement-time curve (dashed line in Fig. 2c) from the reference test was obtained by applying a point-tracking MATLAB algorithm on the test footage. To find the time of first contact t_{c1}, we first obtained the ΔP-time curve of each cycle by subtracting the pressure response of each cycle with the reference response, then we used a script that detects abrupt changes in slope to find t_{c1} on the ΔP-time curve. The t_{c1} from the last 7 cycles out of the 8 cycles in total were plotted for each h in Fig. 2c.

Shape sensing

Four rectangular objects with the same height of 40 mm and the same length of 100 mm, and widths of 40 mm, 60 mm, 80 mm and 100 mm were printed with PLA filament on a FFF 3D printer. The test setup was the same as that in section Size Sensing with Soft Gripper, except that we used a 0-100 kPa pressure sensor (MPX5100DP, NXP) and a $\pm$ 34.5 kPa pressure sensor to measure the pressure in the air tanks and soft gripper, respectively. All control and measurement were done on the data acquisition card with custom software. One actuation cycle was performed for each rectangular object. For each object, the actuation cycle started by setting the proportional pressure regulator at 80 kPa in 20 s, waiting for 15 s to charge the three 0.1 L air tanks, switching on the first valve to disconnect the tanks from the proportional pressure regulator and waiting for 5 s, switching on the second valve to charge the gripper for 60 s, then switching off the second valve to discharge the gripper, and finally switching off the first valve. The ΔP-time curves in Fig. 2e were obtained by subtracting the pressure response of each object with the reference response of empty gripping.

Stiffness sensing

To prepare the soft samples, the pre-polymer components of silicone (A and B, Ecoflex OO-10, Smooth-On) were mixed with a ratio of 1A:1B and degassed. The silicone mixture was then molded in a plastic Petri dish (diameter: 86 mm) to which a thin layer of release agent was applied (Ease release 200, Smooth-On). The silicone was cured at ambient temperature for 4 hours and then taken out of the Petri dish. The silicone sample had a thickness of 17 mm. A laser-cut acrylic plate with a thickness of 8 mm was used as the rigid sample. For both the soft and rigid samples, the single PneuNet actuator was placed horizontally and 5 mm above the sample, with the bottom surface of the actuator parallel to the top surface of the sample. The test setup was the same as that in section Pressure Control with Pressure Measurement.

One actuation cycle was performed for each sample. The actuation cycle started by setting the proportional pressure regulator at 49.9 kPa, waiting for 5 s to charge the 0.1 L air tank, switching on the first valve to disconnect the tank from the proportional pressure regulator and waiting for 10 s, switching on the second valve to charge the actuator for 60 s, then switching off the second valve to discharge the actuator. The ΔP-time curves in Fig. 2g were obtained by subtracting the pressure response of each sample with the reference response of free actuation.

Profile scanning

Two plates (100 mm $\times$ 25 mm $\times$ 15 mm, length $\times$ width $\times$ height) with the top surface in the shape of sine and triangle waves, respectively, were printed with PLA filament on a FFF 3D printer. The single PneuNet actuator was mounted on a robotic arm (Universal Robots UR5e) through a 3D-printed adapter. The actuation system included, in the direction of air flow, a proportional pressure regulator, a 3/2-way solenoid valve, a 0.1 L air tank, and another 3/2-way solenoid valve. The input port of the proportional pressure regulator was connected to the compressed air source that was set at 150 kPa by an independent wall-mounted pressure regulator. The connections of the valves were the same as those described in section Pressure Control with Pressure Measurement. The inlet port of the second valve was connected directly to the actuator through a 2 m long polyurethane air hose (PUN-6X1-BL, Festo). We used a 0-100 kPa pressure sensor (MPX5100DP, NXP) and a 0-50 kPa pressure sensor (MPX5050DP, NXP) to measure the pressure in the air tank and actuator, respectively. All control and measurement were done on the data acquisition card with custom software. We connected the analog input of the robotic arm control board to the 5 V digital output of our data acquisition system and wrote a program in PolyScope that starts the movement of the robotic arm when the analog input changes from 0 V to 5 V. The robotic arm moves horizontally in the mode of MoveL for a total distance of 100 mm at a speed of 2 mm/s. To begin the test, we first manually ran the PolyScope program

on the robotic arm. Since the 5 V digital output of our data acquisition system was initially off, the robotic arm first stayed still. Then we ran a script in our custom software that started the actuation process by setting the proportional pressure regulator at 49.9 kPa and waiting for 5 s, then switching on the first valve to charge the 0.1 L air tank for 10 s, switching off the first valve and waiting for 10 s, then switching on the second valve to charge the actuator for 60 s, then switching on the 5 V digital output to initiate the robotic arm movement and waiting for 90 s, and finally switching off the second valve to discharge the actuator for 30 s. A reference pressure-time response was also measured using the same test procedure without scanning any object. We obtained the ΔP-time curve by substracting the pressure-time response of the scan with the reference pressure-time response. To reconstruct the profile, a calibration ΔP-h curve was first obtained using the same setup. With the calibration curve, the ΔP-time curve from the scan could then be converted into a tip-plate distance-time curve. Finally the time data was converted to x coordinates through the robotic arm moving speed. The ground truth of the profiles were obtained by digital image analysis of flat-bed scanned pictures of the printed objects.

Retroffiting the fluidic sensing approach

Suction cup

To prepare the gripping samples with different stiffness, we molded Ecoflex OO-50, OO-30, OO-10, and GEL 2 (OOO-34) in Petri dishes using the same procedure described in section Stiffness Sensing. The actuation system (Fig. S27) for the suction gripper included, a proportional pressure regulator (-100 kPa to 100 kPa, VEAB-L-26-D13-Q4-V1-1R1, Festo), a 3/2-way solenoid valve, a 15 ml air tank made from an empty CO_2 canister and another 3/2-way solenoid valve. The vacuum input port of the proportional pressure regulator was connected to a vacuum source that was set at -65 kPa by an independent vacuum pressure regulator. We added a 0.75 L air tank as a buffer in between the proportional pressure regulator and the first valve. For the

first valve, the inlet port was connected to the proportional pressure regulator and the normally closed port was connected to the 15 ml air tank. The normally open port was blocked by a cap (FTLLP-6005, Nordson). For the second valve, the inlet port was connected to the suction gripper and the normally closed port was connected to the 15 ml air tank. The normally open port was connected to the atmosphere. We used two $\pm$ 103.4 kPa pressure sensors to measure the pressure in the 15 ml air tank and suction gripper, respectively. We added a flow resistor with an equivalent flow resistance around $7.3 \times 10^9\ \mathrm{Pa} \cdot \mathrm{s}/\mathrm{m}^3$ in between the second valve and the gripper to avoid any dynamic effect during the actuation of the gripper. All control and measurement were done on the data acquisition card with custom software. For each gripping object, two sets of initial vacuum pressure (-62 KPa and - 21 kPa) in the 15 ml air tank were tested. A total of 5 actuation cycles were performed for each test. Each actuation cycle started by setting the proportional pressure regulator and waiting for 15 s, switching on the first valve to vacuum the 15 ml air tank for 15 s, switching off the first valve and waiting for 15 s, then switching on the second valve to vacuum the suction gripper for 60 s, and finally switching off the second valve and waiting for 10 s. At the beginning of each test, the suction gripper was gently pressed on top of the gripping sample to ensure successful gripping upon vacuum. For each actuation cycle, the equilibrium pressure was averaged between 8 s and 10 s after switching on the second valve. For each gripping sample, the equilibrium pressure from the last 4 actuation cycles were plotted in Fig. 4d. The pressure-volume curves (Fig. 4c and Fig. S9) of the suction-based soft gripper when gripping different Ecoflex samples were characterized using the experimental setup in Fig. S28. A total of 5 actuation cycles were performed for each gripping sample. The test started by setting the proportional pressure regulator and waiting for 15 s. Each actuation cycle included switching on the valve to vacuum the gripper for 30 s, then switching off the valve and waiting for 30 s.

To correlate the pressure response of the suction gripper with the pulling force, we started

pulling the suction gripper away from the gripping sample (Ecoflex OO-30) on a custom tensile test setup, after the suction gripper and the 15 ml air tank reached equilibrium pressure. The actuation system for the suction gripper was the same as mentioned above and the suction gripper was connected to a load cell (FLLSB200, FUTEK) through a nylon wire on the custom tensile test setup. The actuation cycle was also the same as mentioned above, except that the second valve was switched off 15 s after it was switched on, disconnecting the suction gripper from the 15 ml air tank and maximizing the sensing resolution, then the load cell moved up vertically on the custom setup at a speed of 1 mm/s. Since the fluidic and tensile test systems were separated, we synchronized both test data based on the detaching moment of the suction gripper.

TPU bending actuator

The TPU bending actuator was designed based on dimensions described in reference (*1*) and printed with TPU filament (Filaflex 82A) on a Fused Filament Fabrication 3D printer (Felix Tec). The actuation system (Fig. S29) included, in the direction of air flow, a proportional pressure regulator (-100 kPa to 500 kPa, VEAB-L-26-D18-Q4-V1-1R1, Festo), a 3/2-way solenoid valve with the normally closed port closed with a cap, a 0.1 L air tank, a second 3/2-way solenoid valve with the normally open port venting to air and the inlet port connected to a flow resistor. In this experiment, we used a different model of the solenoid valve (VDW250-5G-1-01F-Q, SMC) that can handle twice higher pressure than the valves used in other experiments. The input port of the proportional pressure regulator was connected to the compressed air source that was set at 400 kPa by an independent wall-mounted pressure regulator. To reduce the pressure fluctuations during regulation, we added a 0.75 L air tank (CRVZS-0.75, Festo) as a buffer between the proportional pressure regulator and the 3/2-way valve. We used a 0-700 kPa pressure sensor (MPX5700DP, NXP) and a $\pm$ 206.8 kPa pressure sensor (SSCDRRN030PDAA5,

Honeywell) to measure the pressure in the air tank and TPU actuator, respectively. A bidirectional flow sensor (HAFBLF0750CAAX5, Honeywell) was added to measure the flow from the air tank to the TPU actuator during actuation and a flow resistor was used to restrict the flow within the range of the flow sensor. A total of 8 actuation cycles were performed for each test. Each actuation cycle started by setting the proportional pressure regulator at 300 kPa and waiting for 20 s, switching on the first valve to isolate the tank from the pressure regulator and waiting for 10 s, then switching on the second valve to inflate the TPU actuator for 60 s, finally switching off the second valve to deflate the TPU actuator and waiting for 60 s before switching off the first valve for recharging the air tank. For each actuation cycle, the equilibrium pressure was averaged between 40 s and 41 s after switching on the second valve. For each cylindrical object, the equilibrium pressure from the last 5 actuation cycles were plotted in Fig. 3i. Note that the sensor offset of the $\pm$ 206.8 kPa pressure sensor was recalculated for each actuation cycle based on the average of the last 1 s measurements before switching on the second valve.

Filament actuator

The filament actuator was fabricated with the gravity-assisted molding approach described in reference (*2*). A 400 mm long steel rod with a diameter of 1.5 mm was coated with liquid silicone (Elite Double 32 fast) and suspended at a 5 degree angle relative to the vertical direction during curing. The filament actuator was cut into a length of 150 mm after curing. One end of the filament actuator was dip coated with extra silicone to form a thicker part that could be mounted on the robotic forearm by piercing with a plastic pin (Fig. S30). The other end of the filament actuator was connected to a tube fitting (FTLL004, Nordson) and coated with extra silicone to ensure airtightness. The angle of the hinge between two arms was measured with a 3D-printed angle ruler to adjust the position of the cylindrical stopper at different angular displacements θ in Fig. 3a-c. The actuation system (Fig. S30) included, in the direction of air flow,

a proportional pressure regulator (-100 kPa to 500 kPa, VEAB-L-26-D18-Q4-V1-1R1, Festo), a 3/2-way solenoid valve with the normally open port closed with a cap, a 500 mm long air pipe (PUN-6X1-BL, Festo), a second 3/2-way solenoid valve with the normally open port closed with a cap and a third 3/2-way solenoid valve with both the inlet port and normally open port venting to air. The input port of the proportional pressure regulator was connected to the compressed air source that was set at 400 kPa by an independent wall-mounted pressure regulator. The 500 mm long air pipe was used as the actuation tank. To reduce the pressure fluctuations during regulation, we added a 0.75 L air tank (CRVZS-0.75, Festo) as a buffer between the proportional pressure regulator and the 3/2-way valve. We used a 0-700 kPa pressure sensor (MPX5700DP, NXP) and a $\pm$ 206.8 kPa pressure sensor (SSCDRRN030PDAA5, Honeywell) to measure the pressure in the air pipe and filament actuator, respectively. A bidirectional flow sensor (HAFBLF0750CAAX5, Honeywell) was added to measure the flow from the air pipe to the filament actuator during actuation and a flow resistor was used to restrict the flow within the range of the flow sensor. A total of 8 actuation cycles were performed for each test. Each actuation cycle started by setting the proportional pressure regulator at 265 kPa and waiting for 10 s, switching on the first valve to pressurize the air pipe for 20 s, switching off the first valve and waiting for 10 s, then switching on the second valve to inflate the filament actuator for 30 s, finally switching off the second valve and switching on the third valve to deflate the filament actuator and waiting for 20 s before switching off the third valve. Two solenoid valves instead of one were arranged between the air pipe and filament actuator to avoid any leakage, because only the inlet port of the valve allows pressure up to 700 kPa while the other two ports only allow up to 100 kPa. For each actuation cycle, the equilibrium pressure was averaged between 28 s and 29 s after switching on the second valve. For each angular displacement θ, the equilibrium pressure from the last 5 actuation cycles were plotted in Fig. 3c. Note that the sensor offset of the $\pm$ 206.8 kPa pressure sensor was recalculated for each actuation cycle based on the

average of the last 0.5 s measurements before switching on the second valve.

McKibben actuator

The McKibben actuator was fabricated with thermoplastic polyurethane (TPU) bladder and polyethylene terephthalate braided sleeve (PTO0.25BK, Techflex PET Overexpanded). The bladder (130 $\times$ 23.6 mm, length $\times$ width at the deflated state) was made by heat sealing two layers of TPU film (Airtech Stretchlon 200, thickness 38 μm) on a modified 3D printer (Fleix Tec). The PET sleeve was cut into a length of 130 mm with a heat cutter (D-65396 Walluf, HSGM GmbH). The actuation system (Fig. S31) was the same as that for the filament actuator. We used a $\pm$ 206.8 kPa pressure sensor (SSCDRRN030PDAA5, Honeywell) and a $\pm$ 103.4 kPa pressure sensor (SSCDRRN015PDAA5, Honeywell) to measure the pressure in the air pipe and McKibben actuator, respectively. A bidirectional flow sensor (HAFBLF0750CAAX5, Honeywell) was added to measure the flow from the air pipe to the McKibben actuator during actuation and a flow resistor was used to restrict the flow within the range of the flow sensor. A total of 8 actuation cycles were performed for each test. Each actuation cycle started by setting the proportional pressure regulator at 140 kPa and waiting for 10 s, switching on the first valve to pressurize the air pipe for 20 s, switching off the first valve and waiting for 10 s, then switching on the second valve to inflate the filament actuator for 30 s, finally switching off the second valve and switching on the third valve to deflate the filament actuator and waiting for 60 s before switching off the third valve. The data processing was the same as that of filament actuator.

Vacuum-powered commercial soft gripper

We mounted the vacuum-powered commercial soft gripper (SG.S74DS70.SDS70.F.G14F.00, Piab) on the robotic arm and kept the robotic arm stationary throughout the gripping test. The actuation system (Fig. S32) was similar to that described in section Size Sensing with Soft

Gripper, except that a vacuum pressure source and 0.1 L air tank were used. We added a bidirectional flow sensor (HAFBLF0750CAAX5, Honeywell) to measure the flow between the air tank and the soft gripper and a flow resistor to restrict the flow within the range of the flow sensor. A total of 8 actuation cycles were performed for each test. Each actuation cycle started by setting the proportional pressure regulator at -60 kPa and waiting for 10 s, switching on the first valve to pressurize the air pipe for 20 s, switching off the first valve and waiting for 10 s, then switching on the second valve to inflate the gripper for 30 s, finally switching off the second valve to deflate the filament actuator and waiting for 30 s to fully discharge. For each actuation cycle, the equilibrium pressure was averaged between 24 s and 25 s after switching on the second valve. For each gripping object, the equilibrium pressure from the last 5 actuation cycles were plotted in Fig. 4h. Note that the sensor offset of the $\pm$ 103.4 kPa pressure sensor was recalculated for each actuation cycle based on the average of the last 1 s measurements before switching on the second valve.

Commercial soft PneuNet gripper

We mounted the commercial soft PneuNet gripper (SFG-FNC3-N5087-S, Soft Robot Technology) on the robotic arm and kept the robotic arm stationary throughout the gripping test. The actuation system (Fig. S33) was similar to that described in section Size Sensing with Soft Gripper, and we added a bidirectional flow sensor (HAFBLF0750CAAX5, Honeywell) to measure the flow between the air tank and the soft gripper and a flow resistor to restrict the flow within the range of the flow sensor. A total of 8 actuation cycles were performed for each test. Each actuation cycle started by setting the proportional pressure regulator at 70 kPa and waiting for 10 s, switching on the first valve to pressurize the air pipe for 20 s, switching off the first valve and waiting for 10 s, then switching on the second valve to inflate the gripper for 60 s, finally switching off the second valve to deflate the filament actuator and waiting for 60 s to fully dis-

charge. For each actuation cycle, the equilibrium pressure was averaged between 50 s and 51 s after switching on the second valve. For each gripping object, the equilibrium pressure from the last 5 actuation cycles were plotted in Fig. 4k. Note that the sensor offset of the ± 103.4 kPa pressure sensor was recalculated for each actuation cycle based on the average of the last 1 s measurements before switching on the second valve.

Modeling the fluidic sensing approach

We develop a basic model based on the interaction of an extension actuator (with a linear stiffness k) with a rigid wall (Fig. S12), to characterize and better understand the dynamics between the air tank and actuator in the proposed sensing strategy. The linear actuator is initially at atmosphere pressure $p_{\text{act}} = p_{\text{atm}}$, while the tank with compressed air starts at $p_{\text{tank}} = p_0$. We take the sensing target as the initial distance L between the tip of the actuator and the wall. The valve between the air tank and actuator is opened at $t = 0$, so that the system will reach the equilibrium pressure p_1. Assuming incompressible and laminar flow between the air tank and actuator, the Hagen-Poiseuille equation that characterizes the flow rate between air tank and actuator can be written as

$$\frac{dV}{dt} = \frac{p_{\text{tank}} - p_{\text{act}}}{r}, \tag{3}$$

where V represents the volume of air that transfers from the air tank to the actuator, r represents the flow resistance between the air tank and actuator, and p_{tank} and p_{act} indicate the absolute pressure in the air tank and actuator, respectively. According to the ideal gas law, we have

$$p_{\text{tank}} v_{\text{tank}} = n_{\text{tank}} RT, \tag{4}$$

and

$$p_{\text{act}} v_{\text{act}} = n_{\text{act}} RT, \tag{5}$$

where R represents the ideal gas constant, T represents the absolute temperature of the air, and v_{tank} and v_{act} represent the internal geometrical volume of the air tank and actuator, respectively. The volume v_{tank} is constant, while v_{act} depends on the interaction of the actuator with its environment. Assuming the internal volume of the actuator does not change anymore after the actuator comes into contact with the wall, we have

$$v_{\mathrm{act}} = \begin{cases} A(l_0 + \frac{(p_{\mathrm{act}} - p_{\mathrm{atm}})A}{k}) & \text{if } \frac{(p_{\mathrm{act}} - p_{\mathrm{atm}})A}{k} < L, \\ A(l_0 + L) & \text{if } \frac{(p_{\mathrm{act}} - p_{\mathrm{atm}})A}{k} \geq L, \end{cases} \tag{6}$$

where A and l_0 represent the cross section and initial length of the actuator, respectively. By substituting equation 6 into equation 5, we have

$$p_{\mathrm{act}} = \begin{cases} \dfrac{-(Al_0 - \frac{p_{\mathrm{atm}}A^2}{k}) + \sqrt{(Al_0 - \frac{p_{\mathrm{atm}}A^2}{k})^2 + 4n_{\mathrm{act}}RT\frac{A^2}{k}}}{\frac{2A^2}{k}} & \text{if } \frac{(p_{\mathrm{act}} - p_{\mathrm{atm}})A}{k} < L, \\ \dfrac{n_{\mathrm{act}}RT}{A(l_0 + L)} & \text{if } \frac{(p_{\mathrm{act}} - p_{\mathrm{atm}})A}{k} \geq L. \end{cases} \tag{7}$$

Combining equations 3, 4 and 5, we obtain two differential equations that describe the change of the amount of air mass n_{tank} in the air tank and n_{act} in the actuator over time

$$\frac{Mdn_{\mathrm{tank}}}{\rho dt} = -\frac{\frac{n_{\mathrm{tank}}RT}{v_{\mathrm{tank}}} - p_{\mathrm{act}}}{r}, \tag{8}$$

$$\frac{Mdn_{\mathrm{act}}}{\rho dt} = \frac{\frac{n_{\mathrm{tank}}RT}{v_{\mathrm{tank}}} - p_{\mathrm{act}}}{r}, \tag{9}$$

where M and ρ represent the molar mass and density of air, respectively, and p_{act} is given by equation 7. With initial conditions $n_{\mathrm{tank}}\Big|_{t=0} = p_0 v_{\mathrm{tank}}/RT$ and $n_{\mathrm{act}}\Big|_{t=0} = p_{\mathrm{atm}}Al_0/RT$, equations 8 and 9 can be solved numerically.

For the parameter values presented in Table S3, we determine the pressure-time and pressure-volume responses, calibration and sensing resolution points as shown in Fig. S12b-e. Note that for the basic interaction of the linear extension actuator with a rigid wall, assuming that the

actuator comes into contact with the wall at the equilibrium state, the initial and the equilibrium internal volume of the actuator are known under different environment settings, i.e., $v_0 = Al_0$, $v_1 = A(l_0+L)$, then we also have $dv_1/dL = A$. Therefore, we can also determine the analytical solution of the equilibrium pressure p_1 and sensing resolution dp_1/dL from equation 1 and 2 ($\xi = L$)

$$p_1 = \frac{p_0 v_{\text{tank}} + p_{\text{atm}} A l_0}{v_{\text{tank}} + A(l_0 + L)}, \tag{10}$$

$$\frac{dp_1}{dL} = -\frac{p_0 v_{\text{tank}} + p_{\text{atm}} A l_0}{(v_{\text{tank}} + A(l_0 + L))^2} A. \tag{11}$$

With the numerical simulation and analytical solution, we can qualitatively study the influence of each individual system parameter on the sensing resolution. The initial internal volume of the actuator can be changed by varying either the initial length l_0 (Fig. S12f), or the cross section A (Fig. S12g), which interestingly have different effects on the sensing resolution. To understand this difference, we can first look at an extreme case when the volume of the actuator is infinitely large. In this extreme case, the air mass in the air tank is too small to extend the actuator, then the sensing resolution is $dp_1/dL = 0$. This vanishing of sensing resolution with an extremely large actuator can be seen from the numerical simulation results when varying either l_0 or A (dp_1/dL becomes zero after l_0 and A exceed a certain value in Fig. S12f and g), but not from the analytical solutions. This is because the analytical solution assumes that the actuator always comes into contact with the wall at the equilibrium state. However, the occurrence of this contact also depends on the initial conditions. Therefore, the numerical simulation results agree with the analytical solution in Fig. S12f and g until the actuator can not reach the wall anymore at given initial conditions. Still, the analytical solution in equation 11 provides theoretical insights on how the sensing resolution is affected by l_0 and A.

Interestingly, the analytical solution also indicates that the stiffness k (Fig. S12h) does not influence the sensing resolution, because k does not affect any of the parameters in equation

11. However, when k is extremely large, the actuator becomes too stiff to extend a distance of L with given initial conditions, such that the sensing resolution becomes $dp_1/dL = 0$ as no contact occurs, which can be seen from the numerical simulation results in Fig. S12h. Therefore, a higher stiffness does require a higher tank and actuation pressure. On the other hand, the magnitude of sensing resolution increases with the initial tank pressure p_0 when the other parameters in equation 11 stay constant(Fig. S12i).

It should be noted that, in this simplified model with the linear extension actuator, we assume that the actuator volume stops changing after it hits the wall. However, in reality, the interaction with the environment does not fully constrain the deformation and thus the internal volume of the actuator, which can complicate the analysis and needs to be analyzed case by case. We believe that, despite the simplifications made in our model, it provides a framework for choosing available parameters for improving the sensing resolution.

Sorting experiment

Four cylinders with the same height of 40 mm and diameters of 40 mm, 60 mm, 80 mm, and 100 mm were printed with PLA filament on a FFF 3D printer. The objects were placed into the input array manually. The soft gripper was mounted on a robotic arm (Universal Robots UR5e) through a 3D-printed adapter and connected to the actuation system via a 3.3 m long polyurethane air hose (TIUB07, SMC) that was winded around the robotic arm. The actuation system (Fig. S34) included, in the direction of air flow, a proportional pressure regulator (-100 kPa to 100 kPa, VEAB-L-26-D13-Q4-V1-1R1, Festo), a normally open 2/2-way solenoid valve (VX243AZ3AAXB, SMC), a 0.4 L air tank (CRVZS-0.4, Festo) and a 3/2-way solenoid valve (VDW250-5G-2-01F-Q, SMC). The input port of the proportional pressure regulator was connected to the compressed air source that was set at 150 kPa by an independent wall-mounted pressure regulator (LRP-1/4-2.5, Festo). The 0.4 L air tank was used as the actuation tank.

To reduce the pressure fluctuations during regulation, we added a 0.75 L air tank (CRVZS-0.75, Festo) as a buffer between the proportional pressure regulator and the 2/2-way valve. We used a 0-250 kPa pressure sensor (MPX4250DP, NXP) and a $\pm$ 34.5 kPa pressure sensor (SSCDRRN005PDAA5, Honeywell, uncompensated pressure up to 40.7 kPa) to measure the pressure in the 0.4 L air tank and soft gripper, respectively. The proportional pressure regulator was connected to the 24 VDC digital output and 10 VDC analog output of the control board of the robotic arm for power and control, respectively. The solenoid valves were connected to the 24 VDC digital outputs of the control board. The pressure sensors were connected to the analog output and analog input of the control board for power and reading, respectively. To establish the connection with the robotic arm (Universal Robots UR5e), we used the urx and pymodbus libraries in Python and ran the code in Jupyter Notebook. The proportional pressure regulator was set at 64 kPa. Since the 2/2-way valve was normally open, the 0.4 L air tank was initially pressurized to 64 kPa. At the beginning of each actuation cycle, the 2/2-way valve was first switched on to isolate the 0.4 L air tank from the proportional pressure regulator. After 0.5 s, the 3/2-way valve was switched on to connect the 0.4 L air tank to the soft gripper. The pressure measurement of the soft gripper started 5 s after the activation of the 3/2-way valve and continued for 0.75 s at a data acquisition frequency of 30 Hz. The algorithm associated the average value of the pressure measurement with the size of the object and used it for the insertion sort afterwards. Compared to the conventional insert sort algorithm, our algorithm inserted objects into a separated sorted array instead of the same input array. All the coordinates of the input and sorted array were preprogrammed in the algorithm. To open the gripper, the 3/2-way valve was first switched off, connecting the gripper to the atmosphere. After 0.5 s, the 2/2-way valve was switched off, reconnecting the 0.4 L air tank to the proportional pressure regulator for charging.

Tomato picking experiment

The tomatoes with stems were bought from a local supermarket. Each tomato was cut off with a remaining piece of the thick stem, which was then inserted into the small slot along a plastic tube without any extra fixture. The plastic tube was clamped vertically at the bottom end to simulate the hanging tomatoes. The soft gripper, actuation system and their connections were the same as those described in section Sorting Experiment. The hanging positions of the three tomatoes and corresponding placing positions were preprogrammed in the python code. The experiment included a dummy actuation cycle, three reference actuation cycles at initial tank pressure $P_0 = 62\text{kPa}, 70\text{kPa}, 78\text{kPa}$, respectively, and three intended actuation cycles for each tomato at $P_0 = 62\text{kPa}, 70\text{kPa}, 78\text{kPa}$, respectively. During the reference actuation cycle, the gripper closed in the air without moving. During the picking actuation cycle, the robotic arm first moved to the preprogrammed picking position and closed the gripper around the tomato. The gripper rotated the tomato 180 degrees and then the robotic arm retracted 8 - 11 cm. For both reference actuation and picking actuation cycles, the pressure measurement started 25 s after the activation of the 3/2-way valve and continued for 1 s at a data acquisition frequency of 30 Hz. The algorithm used the average pressure values at the same P_0 to calculate the ΔP. The ΔP threshold for successful picking was set at 0.2 kPa.

Tomato ripeness sensing experiment

All tomatoes were bought from a local supermarket and we put one tomato inside a zipper bag together with a banana for one week to overripe it. The calibration dummies with a height of 48 mm and diameters (in the horizontal plane) of 45, 50, 55, 60, 65 mm were printed with ABS filament on a Fused Filament Fabrication 3D printer (Ultimaker 3). The actuation system (Fig. S35) was slightly different from that described in section Vacuum-Powered Commercial Soft Gripper. We used a 2 L air tank in between the proportional pressure regulator and the

first valve in order to decrease the time it takes for the tank to fully stabilize at the set pressure using the pressure regulator. We put the flow resistor (7.3×10^9 $\mathrm{Pa} \cdot \mathrm{s/m^3}$) before the second valve instead of after it so that the venting doesn't go through the flow resistor and the gripper opens faster. The positions of the calibration dummies and tomatoes were preprogrammed in the python code. The experiment included one calibration round and five sensing rounds. A data acquisition frequency of 150 Hz was achieved by directly connecting the robotic arm to a laptop through Ethernet cable, while all other experiments with the robotic arm in this work were done with wifi connection. In the calibration round, we performed a reference measurement where the gripper closed in the air at the beginning and performed four gripping events on each dummy, the first of which was to align the dummy inside the gripper and the rest of which were used for calibration. In each sensing round, we performed a reference measurement and two gripping events on each tomato. Between sensing rounds, we shuffled the tomatoes randomly to test the robustness of the sensing strategy. For both calibration and sensing cycles, the tank pressure was set at -69.36 kPa.

	Initial tank pressure p_0 (kPa, absolute)	Tank internal Volume v_{tank} (ml)	Initial gripper pressure p_{atm} (kPa, absolute)	Initial gripper internal volume v_0 (ml)	Equilibrium pressure p_1 (kPa, absolute)	Equilibrium gripper internal volume v_1 (ml)	dv_1/dD (ml/mm)	Calculated dp_1/dD (kPa/mm)	Measured dp_1/dD (kPa/mm)
Piab gripper, D = 60 mm	41.55	100	101.325	47.9	66.9	34.79	0.16	-0.08	-0.08
SMT gripper, D = 60 mm	170.88	300	101.325	96.9	141.49	131.61	-0.13	0.04	0.04

Table S1: **Comparison of the two commercial grippers.** The dv_1/dD is obtained from the linear fits in Fig. S11c and f. The calculated dp_1/dD is based on equation 2. The measured dp_1/dD is obtained from the linear fits of the data in Fig. 4h and k.

Pressure sensor	Pressure range	Accuracy	Price (in Euros)
NPC-1220 series	34 to 689 kPa	± 0.1 %	44
Nidec P-2000 series	49 to 981 kPa	± 0.12 %	20-30
Honeywell ABP series	6 kPa to 1 MPa	± 0.25 %	20-30
Honeywell SSC series	160 Pa to 1 Mpa	± 0.25 %	50-80
NXP MPX5100 series	100 kPa	± 2.5 %	23

Table S2: **Comparison of selected commercial analog pressure sensors.** Prices are according to Mouser Electronics in 2023.

Parameter	Value	Unit
M, molar mass of air	0.02896	kg/mol
ρ, density of air	1.2922	kg/m³
R, ideal gas constant	8.314	J/(mol·K)
T, temperature of the air	273.15	K
v_{tank}, internal geometric volume of the air tank	0.0001	m^3
p_0, initial tank pressure, absolute	170000	Pas
r, flow resistance	5.7139E+08	pas·s/m^3
l_0, initial length of the actuator	0.05	m
A, cross section of the actuator	0.0004	m^2
p_{atm}, atmosphere pressure	101325	Pas
k, actuator stiffness	400	N/m

Table S3: **Parameter values used in the fluidic sensing model.**

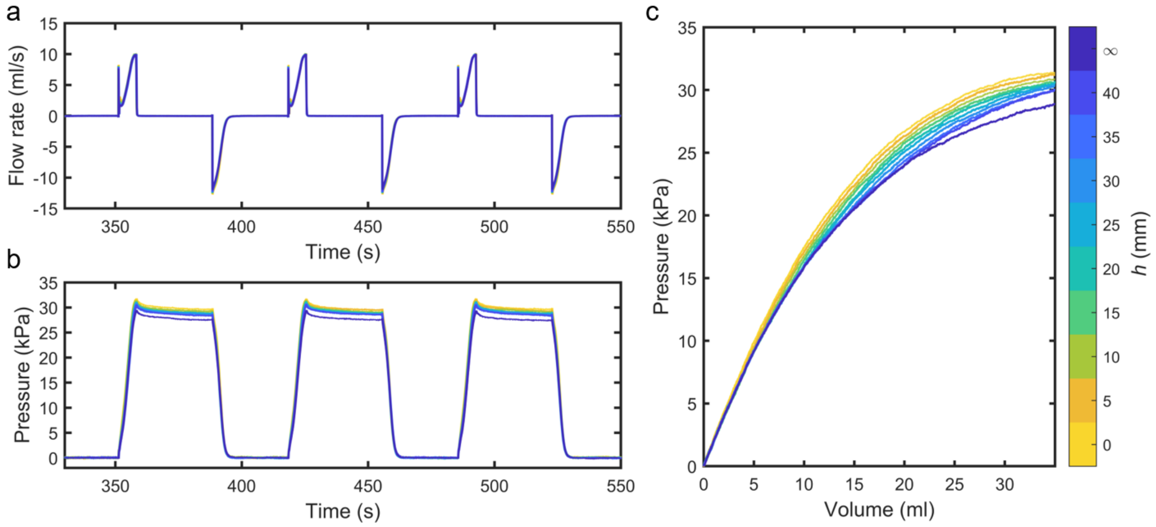

Figure S1: **Characterization of pressure-volume curves of the soft PneuNet actuator bending onto a rigid plate from different heights *h* through controlled flow input. a,** Flow rate of the flow in (positive) and out (negative) of the PneuNet actuator during the last three actuation cycles. **b,** Pressure of the PneuNet actuator during the last three actuation cycles. **c,** Pressure-volume curves of the PneuNet actuator from the last actuation cycle.

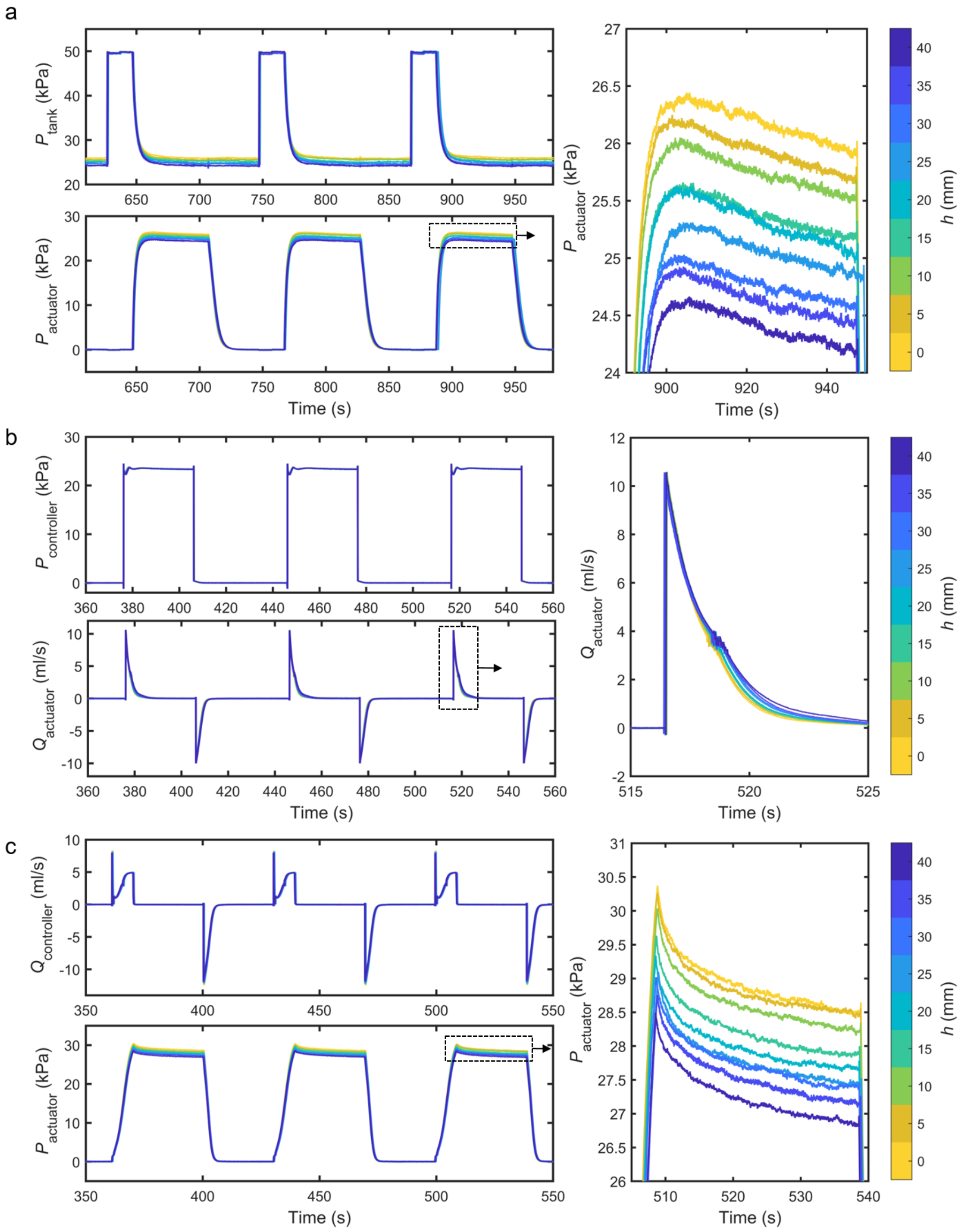

Figure S2: **Experimental data for the fluidic response calibrations with the three fluidic sensing mehtods. a,** Control the total mass using a pressurized air tank and measure the pressure response. **b,** Control the pressure and measure the volume flow input. **c,** Control the volume flow input and measure the pressure. Positive and negative flow rate values represent the flow in and out of the actuator, respectively. For each method, a total of eight actuation cycles were performed at each h and measurements from the last three cycles are shown here.

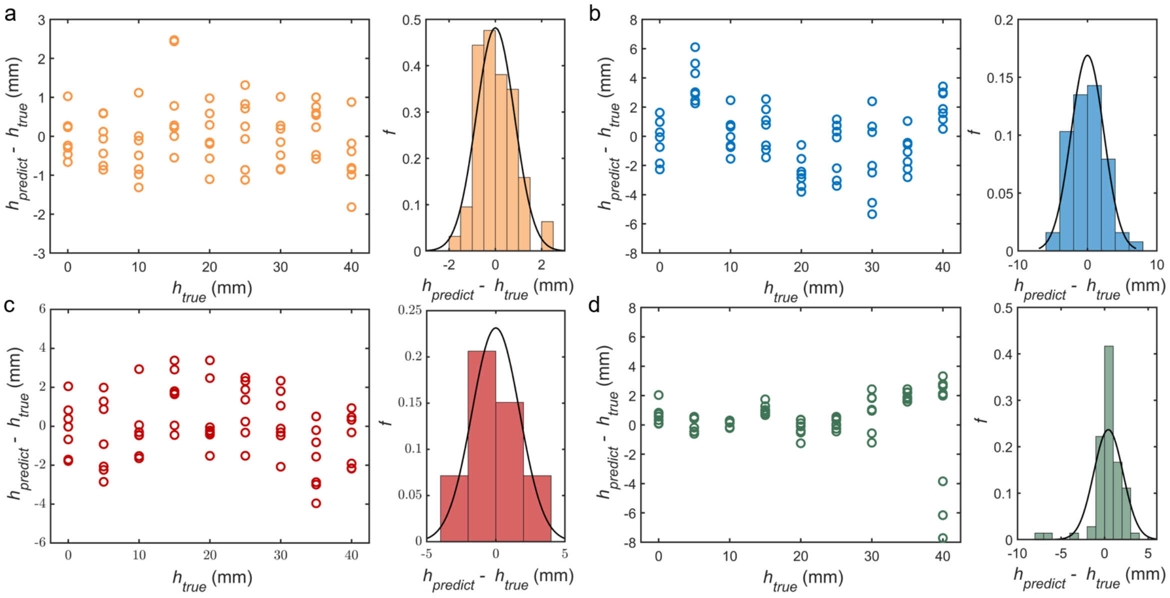

Figure S3: **Accuracy of the various sensing approaches.** The sensing accuracy is determined by the difference between the ground truth (set distance h) and the predicted value of h based on the calibration curves. The probability density function estimate f is shown in histograms with a normal distribution. **a,** Accuracy obtained when controlling the total mass using a pressurized air tank and measuring the pressure response. The h_{predict} is calculated from the calibration curve in Fig. 1b at given pressure measurements. The sensing accuracy of this method is ± 1.7 mm with a 95% confidence interval. **b,** Accuracy obtained when controlling the pressure and measuring the volume flow input. The h_{predict} is calculated from the calibration curve in Fig. 1c at given volume measurements. The sensing accuracy of this method is ± 4.7 mm with a 95% confidence interval. **c,** Accuracy obtained when controlling the volume flow input and measuring the pressure. The h_{predict} is calculated from the calibration curve in Fig. 1d at given pressure measurements. The sensing accuracy of this method is ± 3.4 mm with a 95% confidence interval. **d,** Accuracy obtained when controlling the total mass using a pressurized air tank and measure the pressure response over time. The h_{predict} is calculated based on the interpolation of vertical displacement curve of actuator in the case of free actuation in Fig. 2c at given time of contact measurements. The sensing accuracy of this method is -2.9 to 3.8 mm with a 95% confidence interval.

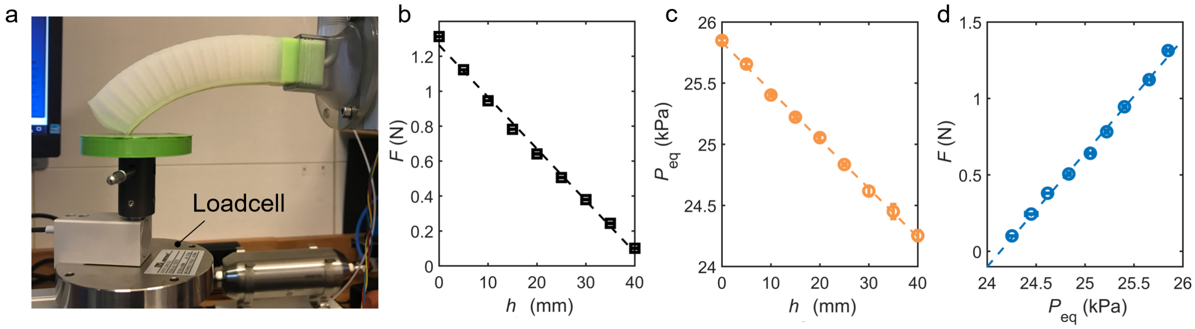

Figure S4: **Force characterization.** **a,** Upon pressurization, the soft PneuNet actuator bends onto a load cell ($\pm$ 100 N, 132151, Instron) from a height h. **b,** Force measured for different initial h. **c,** Equilibrium pressure observed for different initial h. **d,** The obtained force-equilibrium pressure calibration curve. The dashed lines in **b-d,** represent a linear fitting. The error bars represent the standard deviation of four measurements.

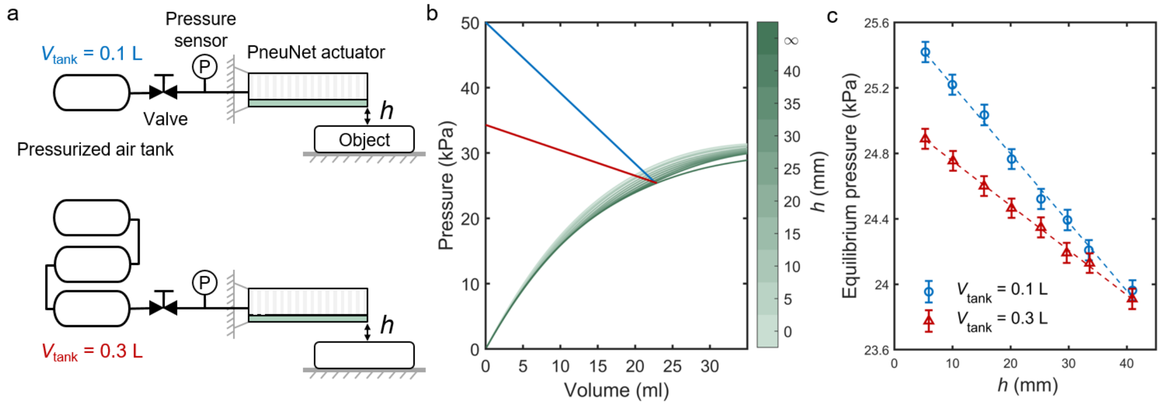

Figure S5: **Tuning the sensing resolution by varying the air tank volume.** **a,** Schematics of the setup with 0.1 L and 0.3 L steel air tanks. **b,** Schematic of the intersections of pressure-volume curves between the actuator and air tanks. The blue and red lines represent the pressure-volume curves of the 0.1 L and 0.3 L air tanks, respectively. **c,** h-pressure calibration curves with 0.1 L and 0.3 L steel air tanks. One actuation cycle was performed at each h, in which the actuator was inflated for 60s. The error bars represent the standard deviation of the non-smoothed pressure sensor (MPX5100DP, NXP) measurements (1000Hz) within the last 5s.

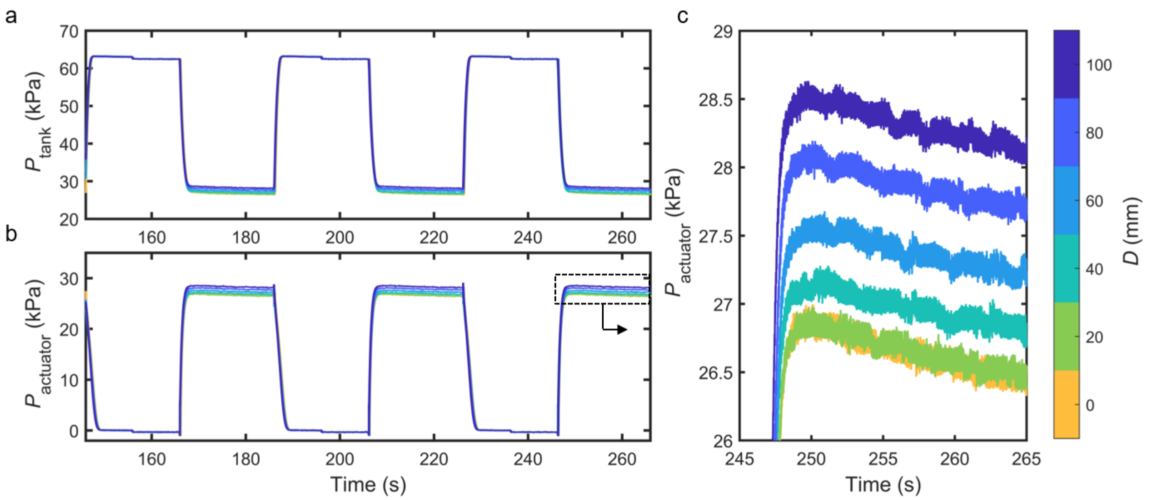

Figure S6: **Experimental data for the *D*-pressure calibrations of the soft gripper. a,** Pressure of the air tank during the last three actuation cycles. **b,** Pressure of the PneuNet actuator during the last three actuation cycles. **c,** Pressure of the PneuNet actuator during the last actuation cycle. The pressure-time curve at $D = 20$ mm almost overlaps with the curve at $D = 0$ mm because the gripper has a lower grasping limit slightly larger than 20 mm and the actuators are barely touching the object with $D = 20$ mm.

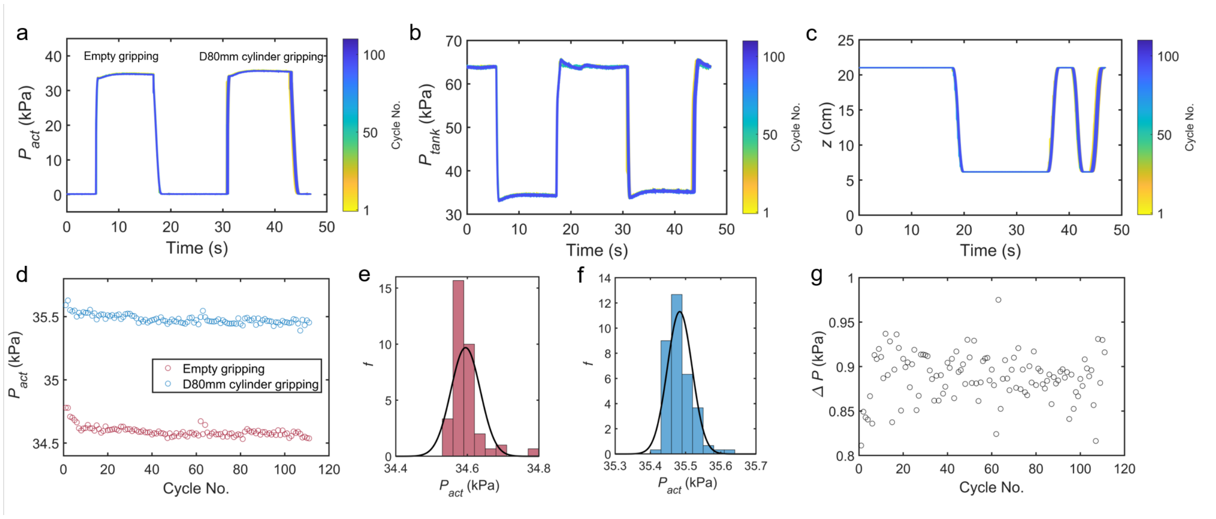

Figure S7: **Cyclic testing of the sensing strategy.** We tested a total of 111 cycles of the soft PneuNet gripper (Fig. 1e) on the UR5 robotic arm. Each cycle contains two gripping events, the first one with nothing inside the gripper, the second one with a 80 mm diameter cylinder inside the gripper. In the second gripping event, after the gripper grasps the object, the robotic arm first moves up and holds the object in the air, then moves down and releases the object before going to the next cycle. For both gripping events, the equilibrium pressure inside the actuator is averaged between 8 s and 9 s after the opening of the valve, which corresponds to the time when the object was held in the air during the second gripping event. **a,** Pressure-time measurements of the actuator from 111 cycles superimposed on top of each other. **b,** Pressure-time measurements of the tank from 111 cycles superimposed on top of each other. **c,** Measurements of the TCP Z coordinate of the robotic arm from 111 cycles superimposed on top of each other. **d,** Equilibrium pressure of the two gripping events for all 111 cycles. **e,** Distribution of the equilibrium pressure of the first gripping event over 100 cycles. The mean value is 34.5951 ± 0.0824 kPa (95% confidence interval). **f,** Distribution of the equilibrium pressure of the second gripping event over 100 cycles. The mean value is 35.4834 ± 0.0706 kPa (95% confidence interval). Note that the soft gripper tested here is a different version from the one tested in Fig. 1 because the original one was accidentally damaged. We slightly changed the actuator design, resulting in a higher actuation pressure. **g,** The pressure difference $\Delta P = P_{act\_D80} - P_{act\_empty}$ between the two gripping events over 111 cycles.

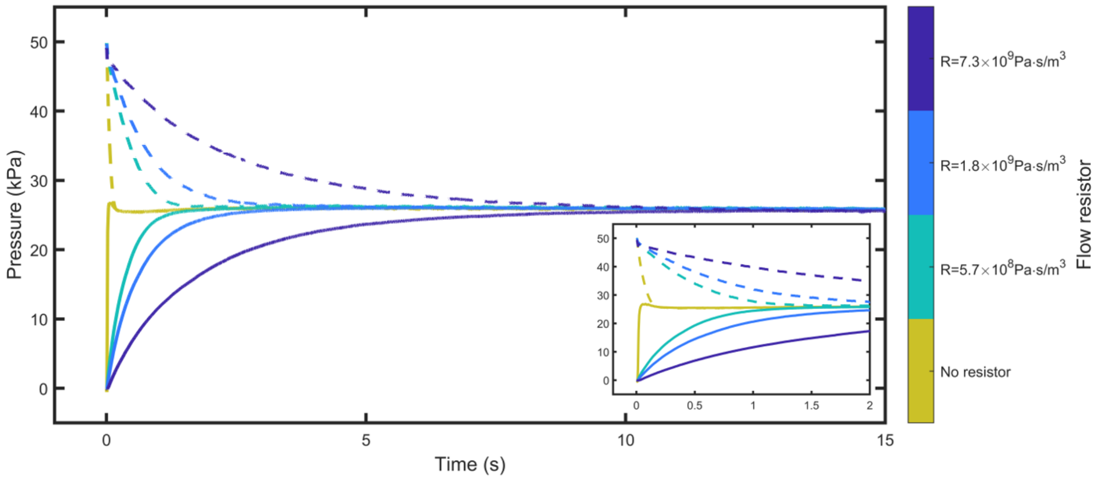

Figure S8: **Tuning the response time with different flow resistors in between the valve and actuator.** The dashed curves represent the pressure in the 0.1 L air tank. The solid curves represent the pressure in the PneuNet actuator. The initial distance h between the actuator and rigid plate was set at 20 mm. Tests were done by switching the flow resistor shown in Fig. S24. The fluidic circuit still has some flow resistances due to the valve and tube connections, even when no flow resistor was added to the circuit. Therefore, the response time can be further improved by reducing these flow resistances in the circuit.

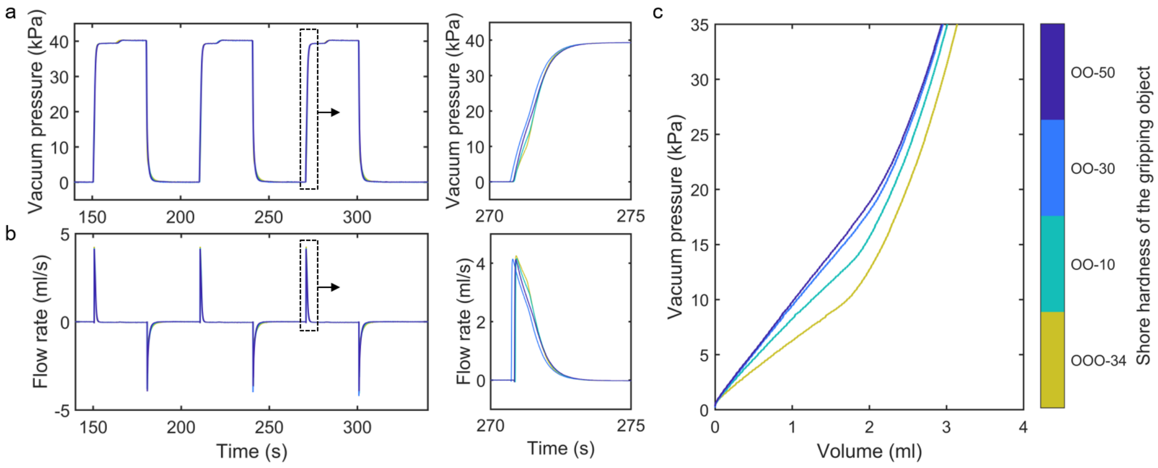

Figure S9: **Characterization of pressure-volume curves of the suction gripper attaching to soft objects with different shore hardness through controlled pressure input. a,** Pressure of the suction gripper during the last three actuation cycles. **b,** Flow rate of the flow going out of (positive) and into (negative) the suction gripper during the last three actuation cycles. **c,** Pressure-volume curves of the suction gripper from the last actuation cycle.

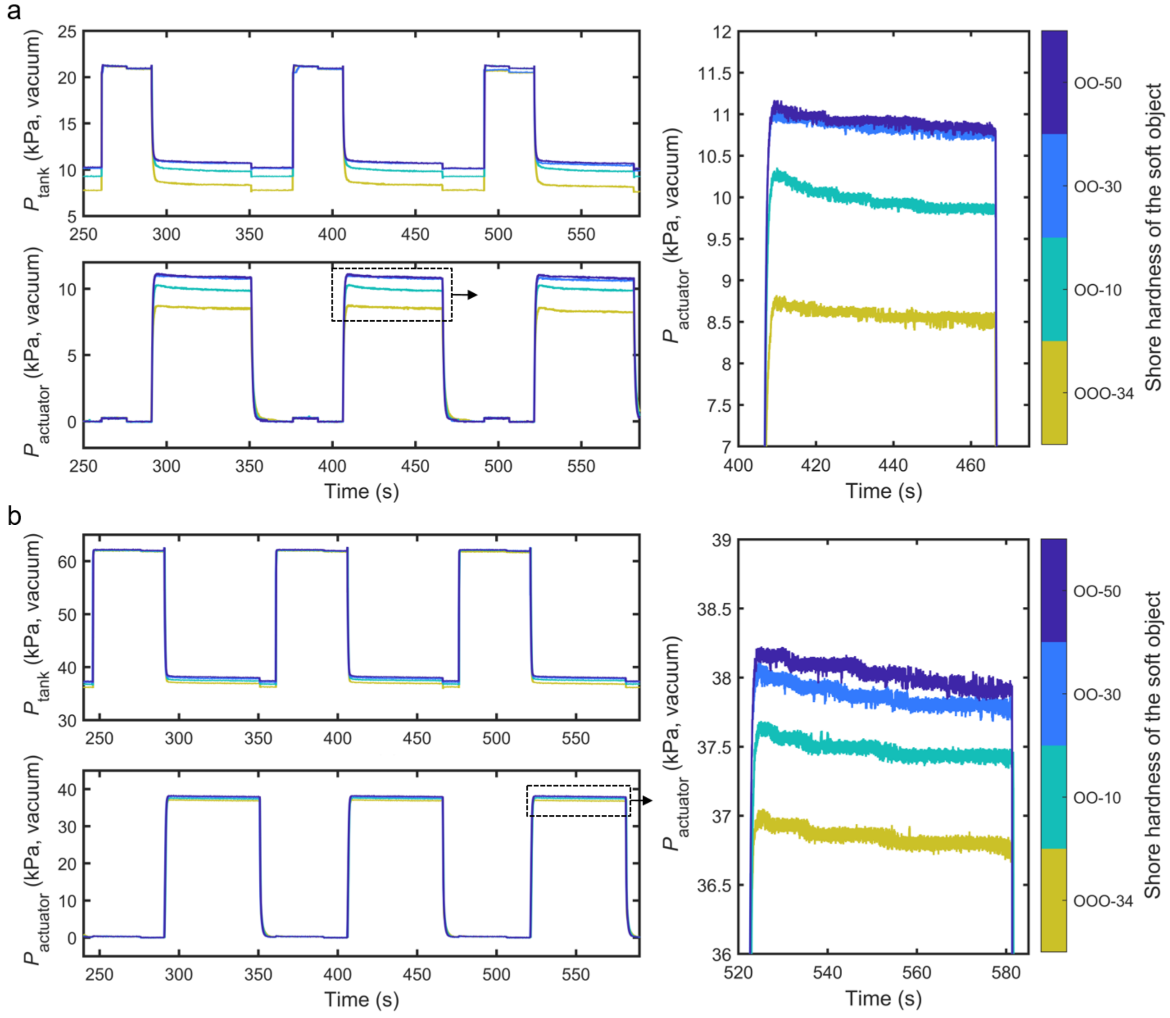

Figure S10: **Experimental data for the stiffness-pressure calibrations of the suction gripper with two different vacuum pressures in the steel air tank. a,** P_0 = 21 kPa (vacuum). **b,** P_0 = 62 kPa (vacuum).

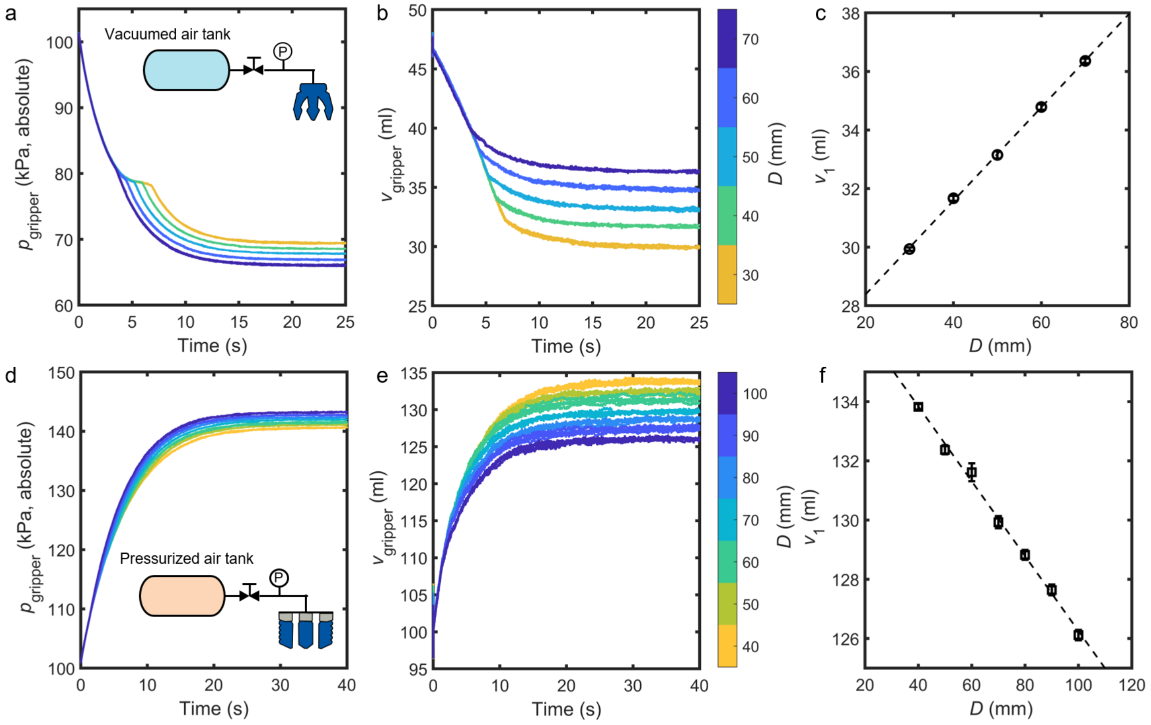

Figure S11: **Internal volume estimation of the commercial grippers when gripping cylindrical objects with different diameters.** The dashed lines in **c and f** represent linear fits of the data. The error bars in **c and f** represent the standard deviation of five measurements.

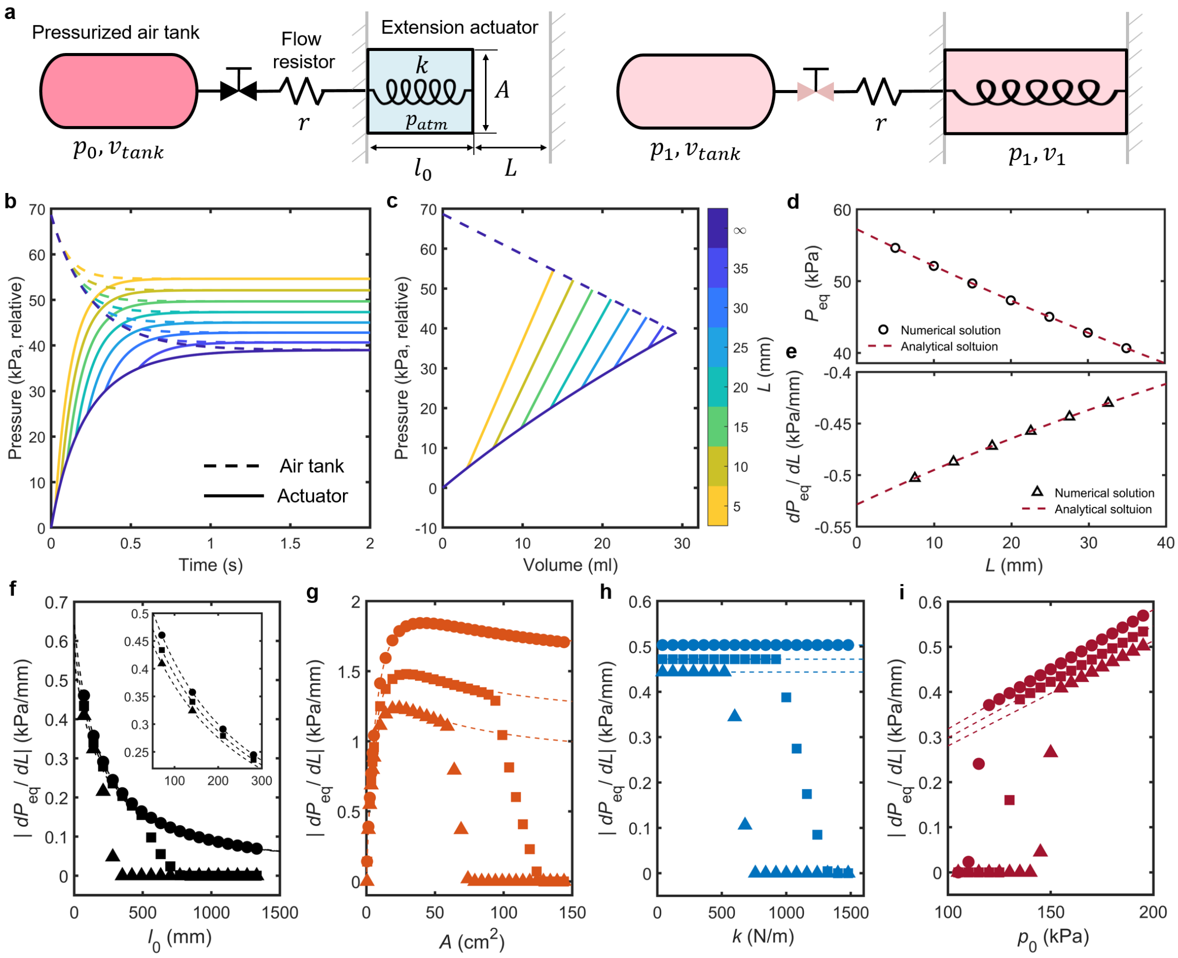

Figure S12: **Modeling the fluidic sensing approach. a,** Schematics of a simplified sensing scenario: an extension actuator with a linear stiffness k comes into contact with a rigid wall from a distance L. **b-e,** Simulation results using the input values shown in Table S3: pressure-time (**b**) and pressure-volume (**c**) curves of the air tank and actuator; calibration curve of P_{eq} over L (**d**); sensing resolution curve of dP_{eq}/dL over L (**e**). **f-i** Simulation results of the effects of actuator's initial length l_0 (**f**), cross section A (**g**), stiffness k (**h**) and initial tank pressure p_0 (**i**) on the sensing resolution magnitude $|dP_{\text{eq}}/dL|$. The makers and dashed lines in **f-i** represent numerical simulation results and analytical solutions, respectively. The circular, square and triangular markers in **f-i** represent $L = 7.5, 17.5, 27.5$ mm, respectively. The results in **f-i** are based on the input values in Table S3 except that an initial tank pressure of 300 kPa (absolute pressure) is used in **g**.

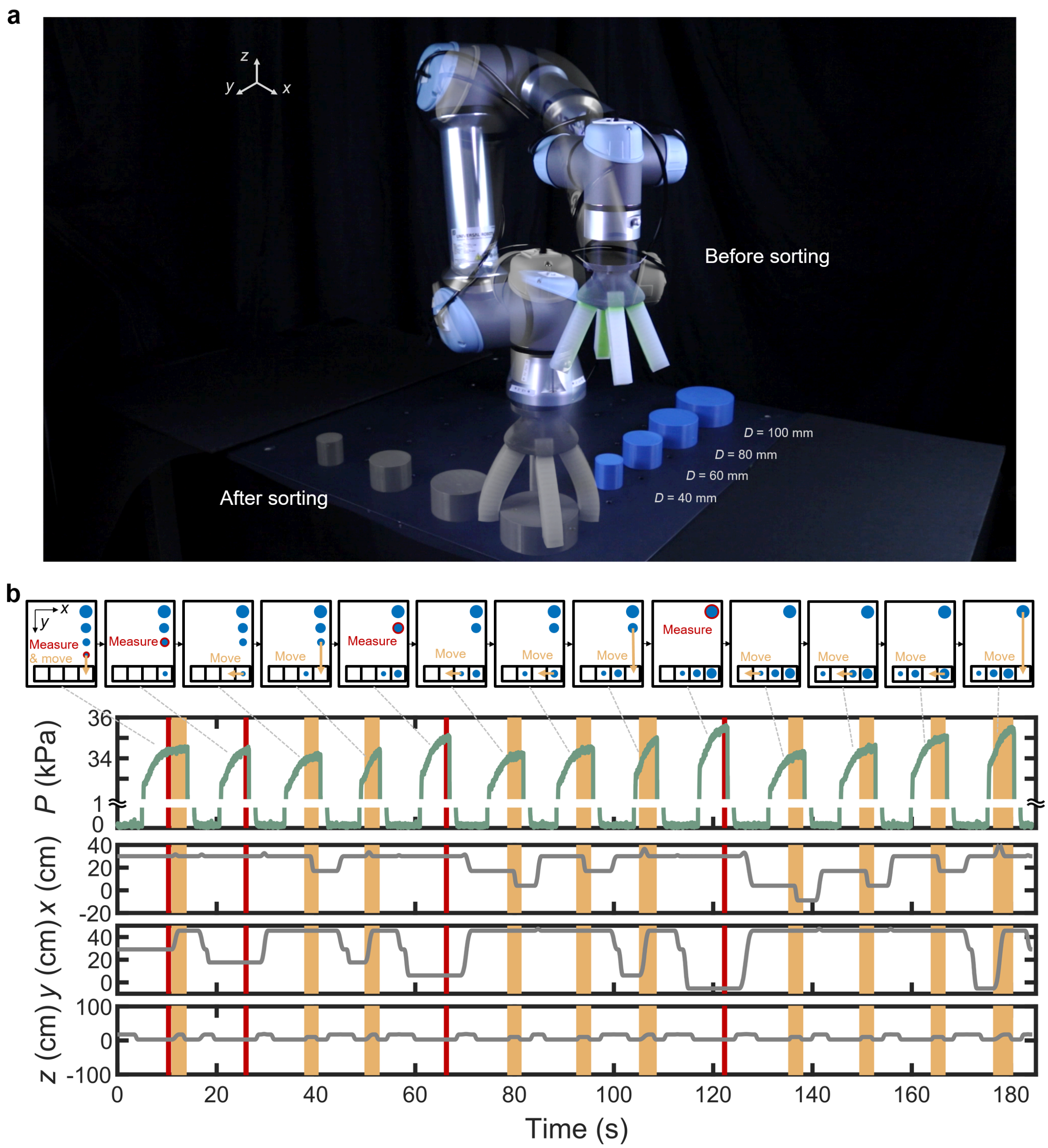

Figure S13: **Sorting experiment from Fig. 5a with a different input order of cylindrical objects with a diameter of 40 mm, 60 mm, 80 mm, 100 mm.**

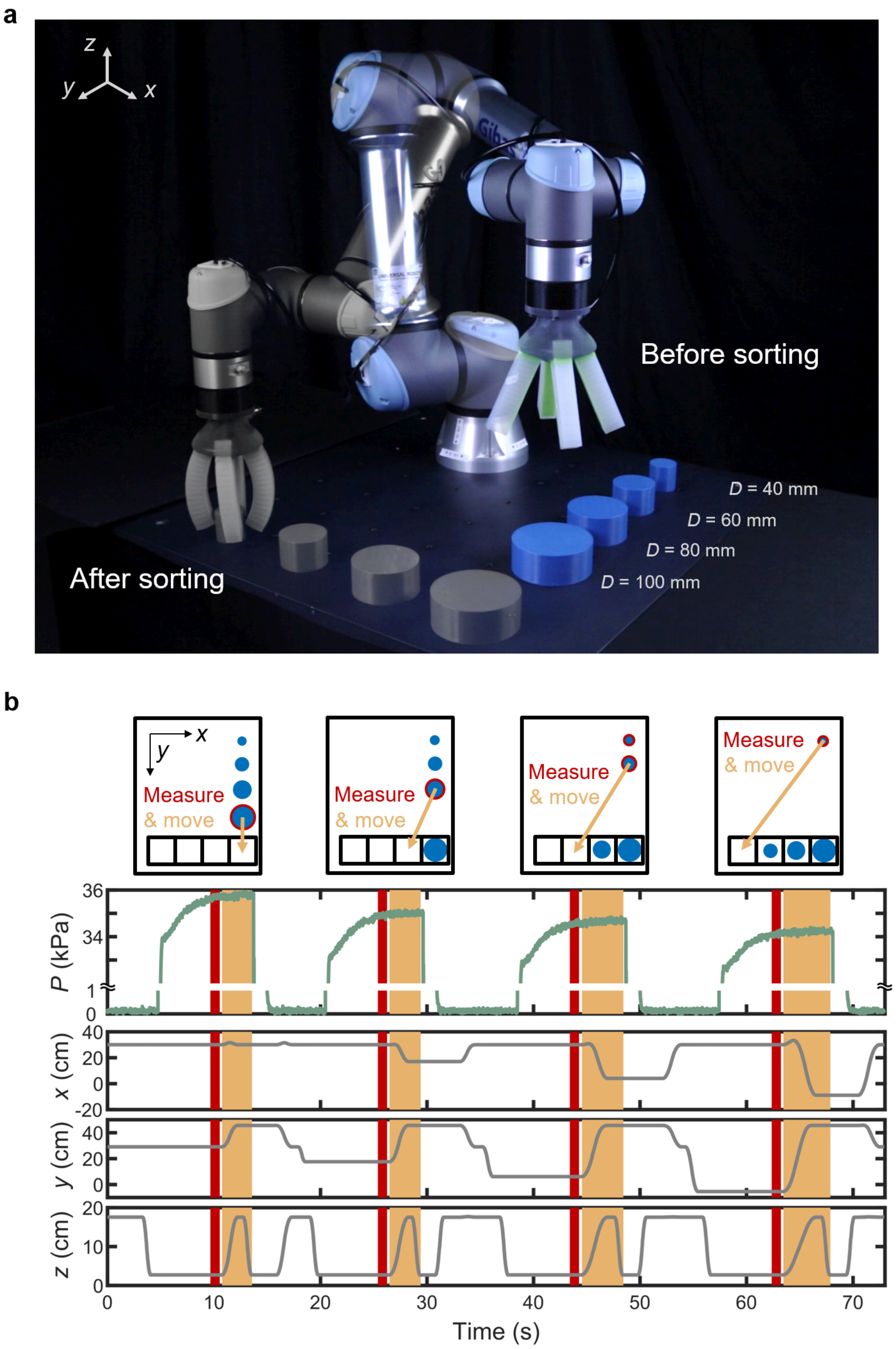

Figure S14: **Sorting experiment from Fig. 5a with a different input order of cylindrical objects with a diameter of 100 mm, 80 mm, 60 mm, 40 mm.**

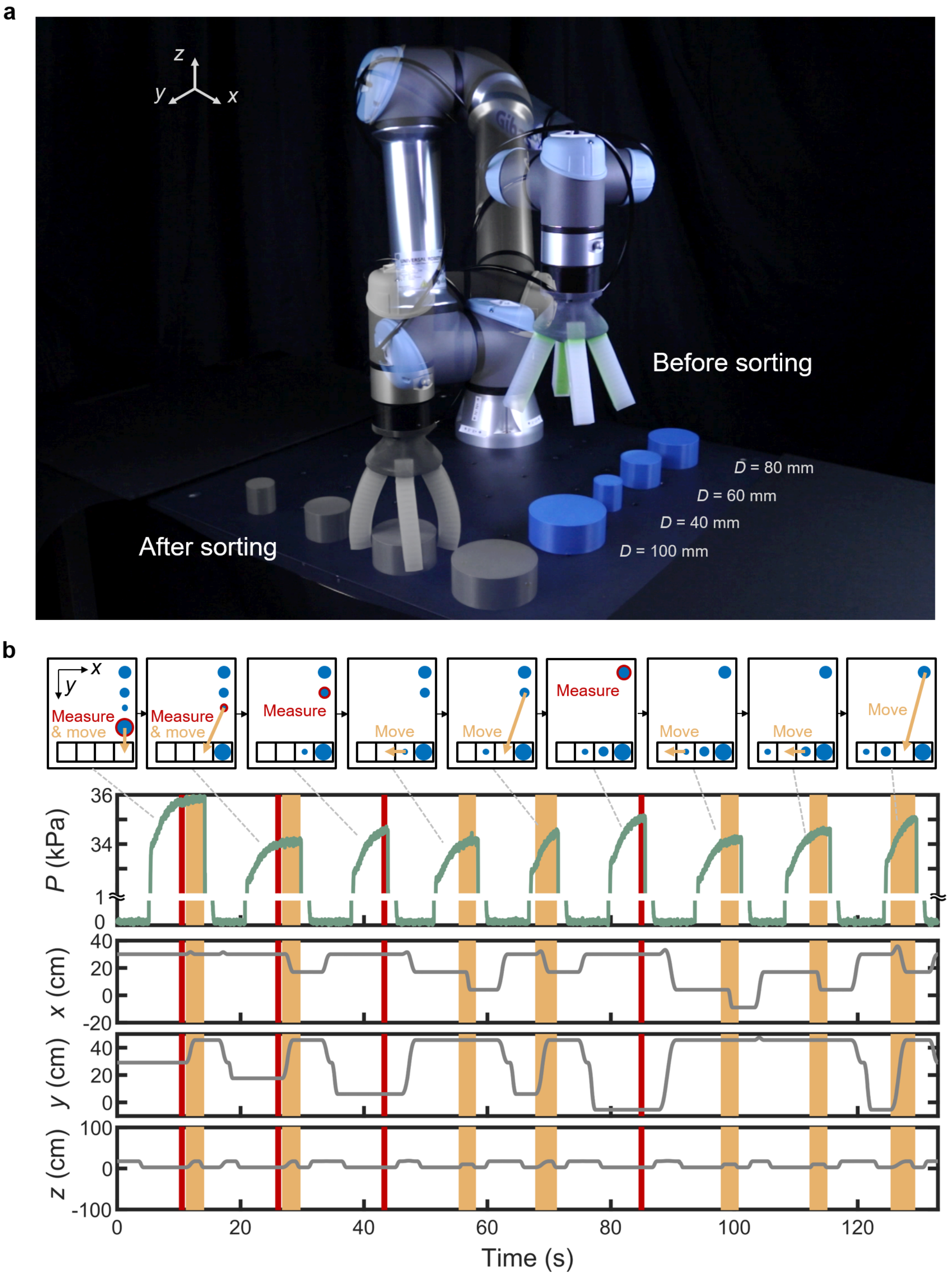

Figure S15: **Sorting experiment from Fig. 5a with a different input order of cylindrical objects with a diameter of 100 mm, 40 mm, 60 mm, 80 mm.**

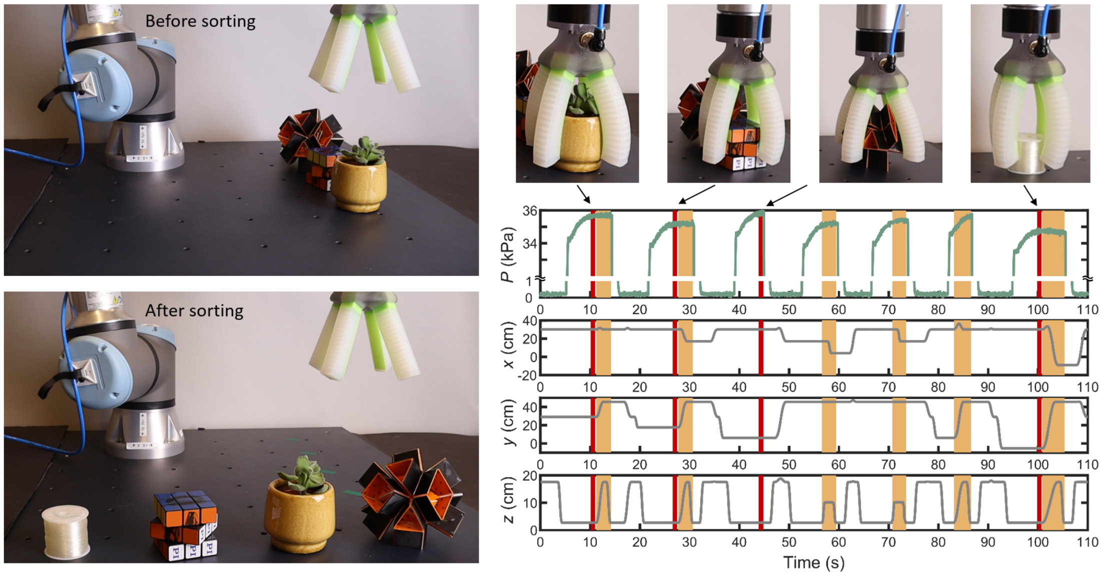

Figure S16: **Sorting experiment with random objects: 1) Small plant. 2) Magic cube. 3) Prismatic metamaterial (*3*). 4) Spool.**

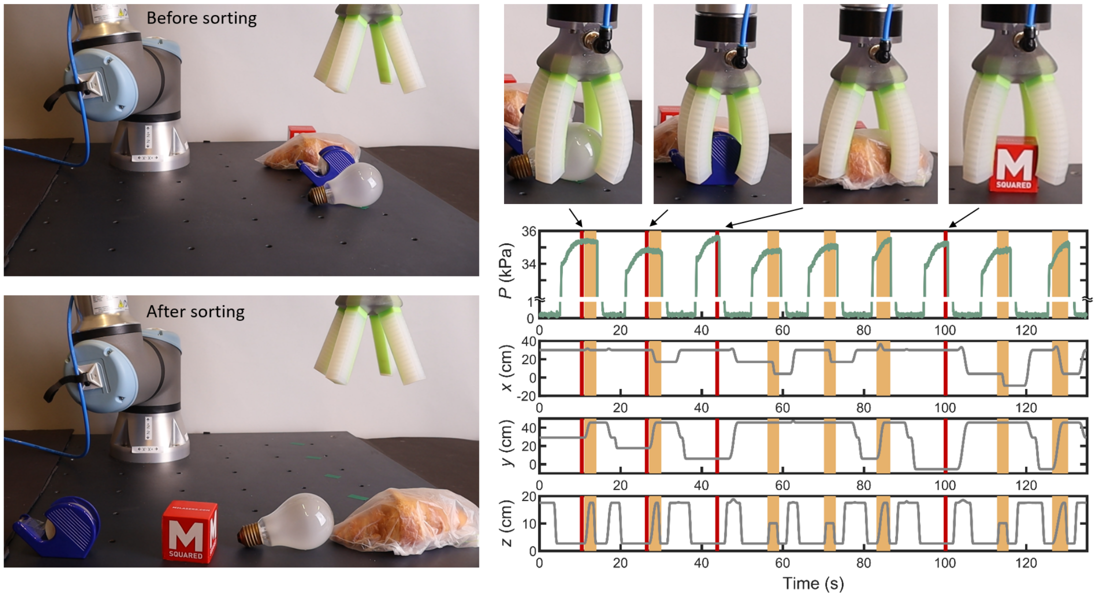

Figure S17: **Sorting experiment with random objects: 1) Light bulb. 2) Tape cutter. 3) Croissant. 4) Stress relief cube.**

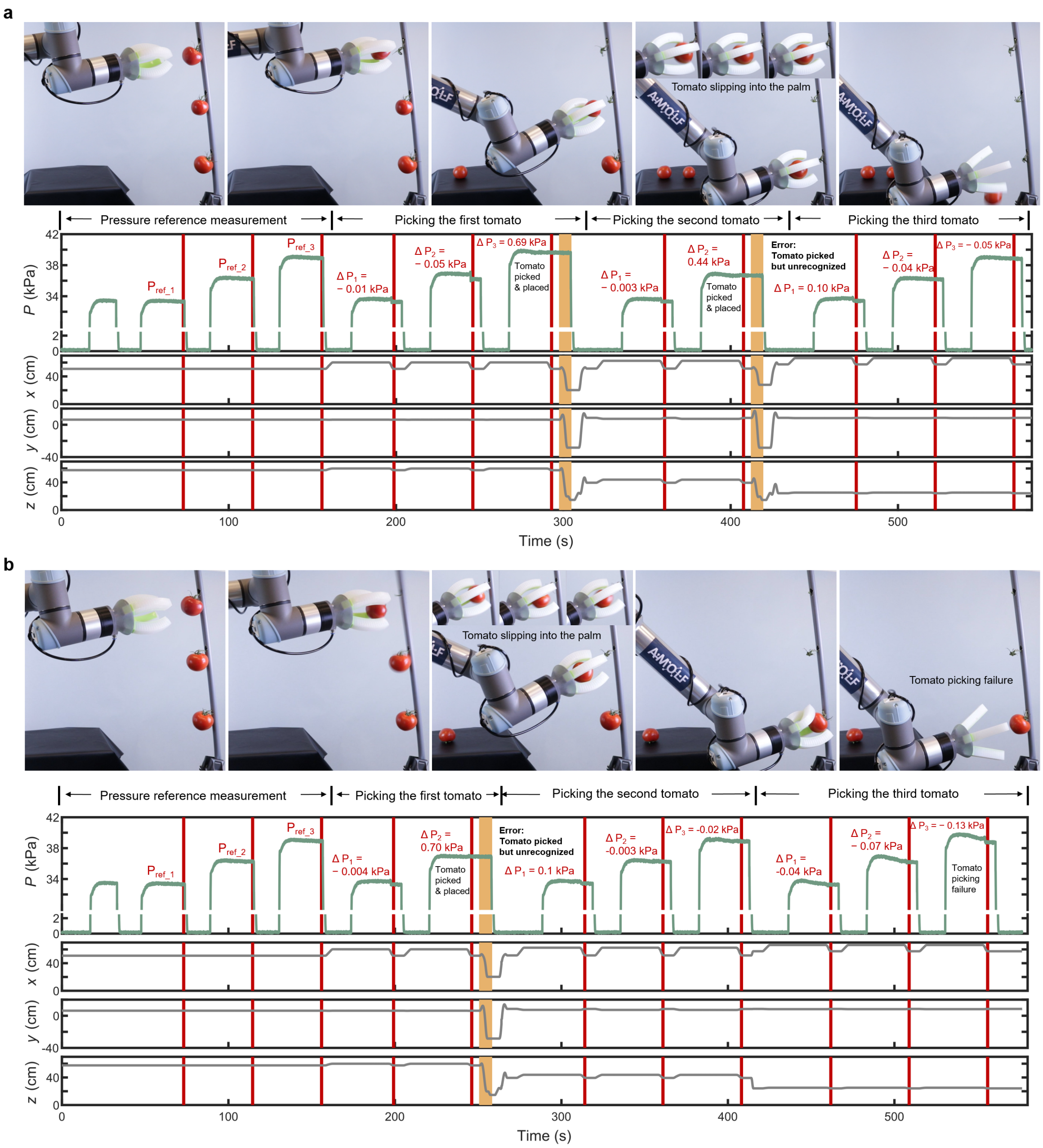

Figure S18: **Snapshots of the tomato picking experiments and gripper pressure and TCP coordinates over time during the picking experiments** (Demo 2 and 3 in Supplementary Video 5). The red band represents pressure feedback measurement, and the yellow band represents tomato placement. During the picking of the third tomato in **a** and the second tomato in **b**, the tomato slipped into the palm of the soft gripper, resulting in $\Delta P = 0.1$ kPa which is smaller than the threshold (0.2 kPa). The tomato picking was not recognized and the gripper dropped the tomato and started a new picking attempt. The gripper failed to pick the third tomato in **b**.

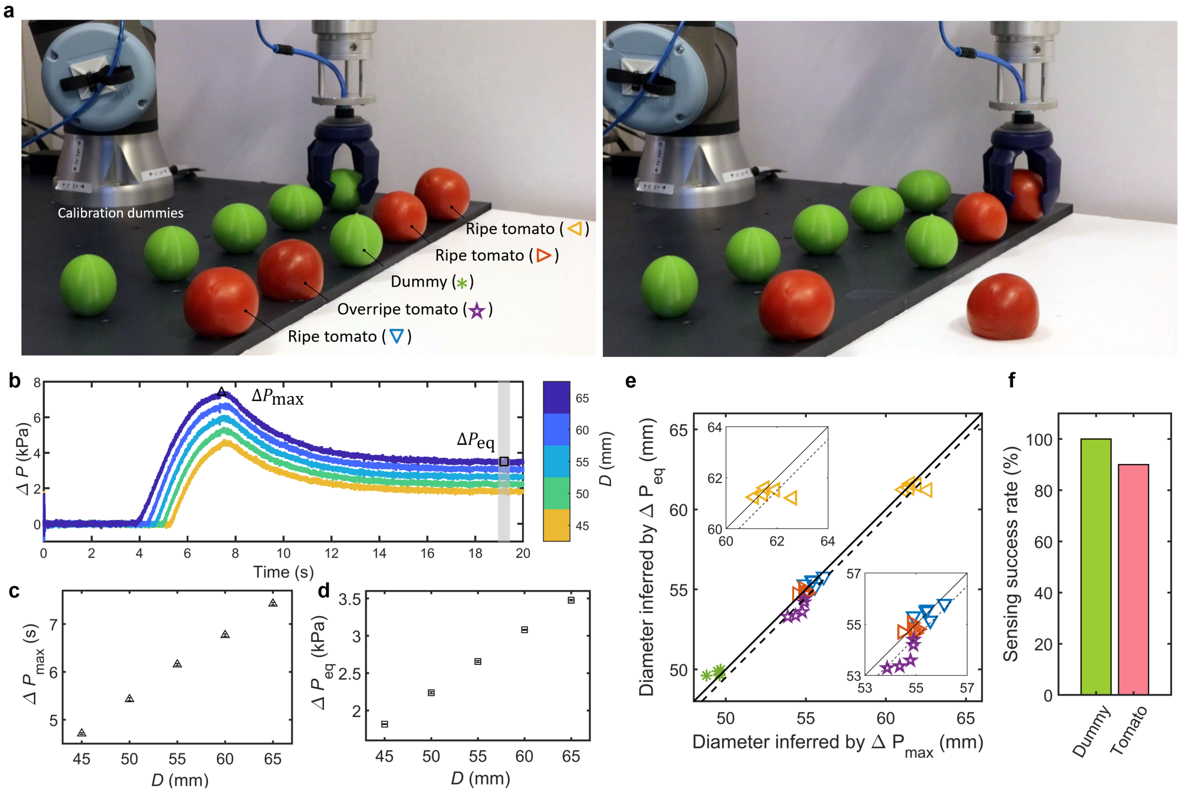

Figure S19: **Picking out the overripe tomato with fluidic sensing based on calibrations with ΔP_{max} and ΔP_{eq}. a,** Snapshots of the closed-loop control demonstration (Demo 2 in Supplementary Video 6). **b-d,** Calibration results. Experimental results from three measurements are plotted for each D in **b**. The error bars in **c,d** represent the standard deviation of three measurements. **f-m,** Sensing results. The solid line in **e** represents that the object diameter inferred by ΔP_{max} equals that inferred by ΔP_{eq}, the dashed line represents the object diameter inferred by ΔP_{eq} is 0.5 mm smaller than that inferred by ΔP_{max}.

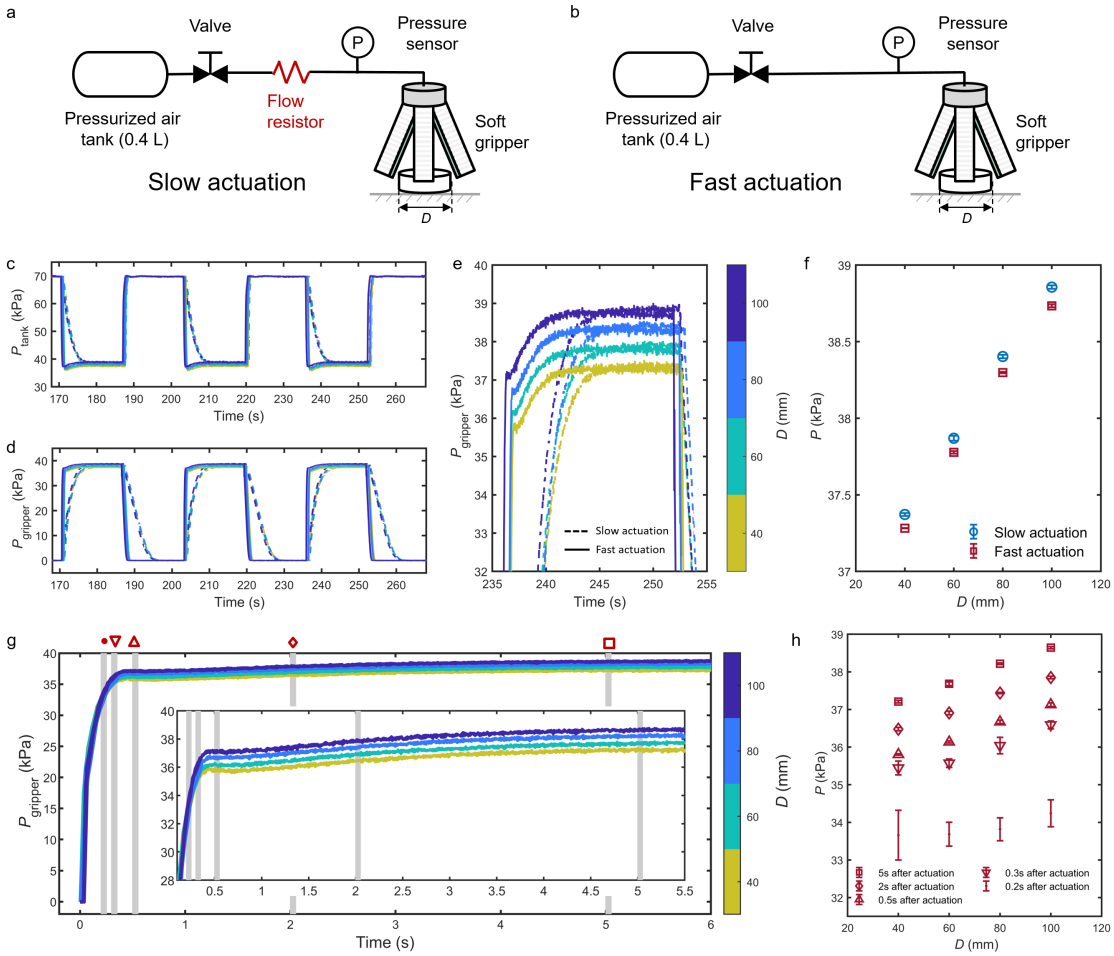

Figure S20: **Influence of the actuation rate on the *D*-pressure calibrations of the soft gripper. a, b,** We vary the actuation rate by placing a flow resistor ($R = 5.7 \times 10^8 Pa \cdot s/m^3$) between the solenoid valve and the gripper. **c-e,** Experimental results of the soft gripper gripping cylindrical objects with a diameter $D = 40$ mm, 60 mm, 80 mm and 100 mm. The dashed and solid curves represent the tests in slow and fast actuation configurations, respectively. A total of eight actuation cycles were performed on each cylindrical object, and the last three cycles are shown in **c** and **d**. **f,** *D*-pressure calibrations in slow and fast actuation configurations. Each data point represents the average value of the equilibrium pressure in the last five actuation cycles. For each actuation cycle, the equilibrium pressure is averaged over a 5 s period starting at 10 s after the actuation. **g, h,** Pressure measurements in fast actuation configuration and *D*-pressure calibrations with different waiting time after the actuation. Results from the last five actuation cycles are superimposed in **g** for each gripping object. Each data point in **h** represents the average value of the gripper pressure in the last five actuation cycles. For each actuation cycle, the pressure in the gripper is averaged over a 0.05 s period starting at 0.2 s, 0.3 s, 0.5 s, 2 s, 5 s after the actuation (the opening of the valve in **b**). The error bar represents the standard deviation of the gripper pressure in the last five actuation cycles.

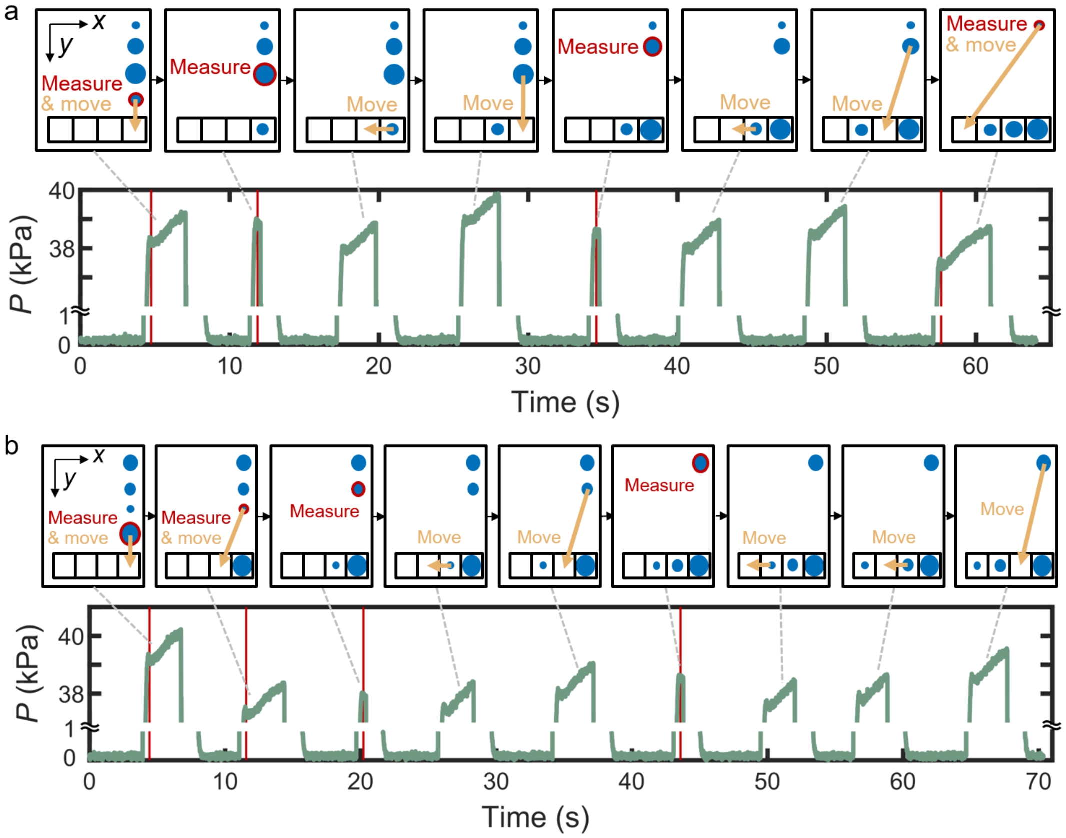

Figure S21: **Sorting experiments with a faster speed.** The input order of cylindrical objects is 60 mm, 100 mm, 80 mm, 40 mm in **a** (Demo 7 in Supplementary Video 4) and 100 mm, 40 mm, 60 mm, 80 mm in **b** (Demo 8 in Supplementary Video 4). For each measurement event, the equilibrium pressure was averaged over a 0.05 s period starting at 0.5 s after the actuation, as indicated by the red bands in the plot. The four pressure feedback measurements (average $\pm$ standard deviation) in **a** are 38.21 ± 0.11 kPa, 38.84 ± 0.07 kPa, 38.59 ± 0.07 kPa, 37.44 ± 0.07 kPa, respectively. The four pressure feedback measurements (average $\pm$ standard deviation) in **b** are 39.17 ± 0.11 kPa, 37.33 ± 0.06 kPa, 37.92 ± 0.07 kPa, 38.52 ± 0.06 kPa, respectively. Note that the initial tank pressure was set at 74 kPa here to ensure successful gripping of each object, especially the one with a diameter of 40 mm. Note that the coordinates of the robotic arm were intentionally not saved in these experiments in order to obtain a high data frequency ($\sim$ 87 Hz) that allows the fast sensing over a 0.05s period.

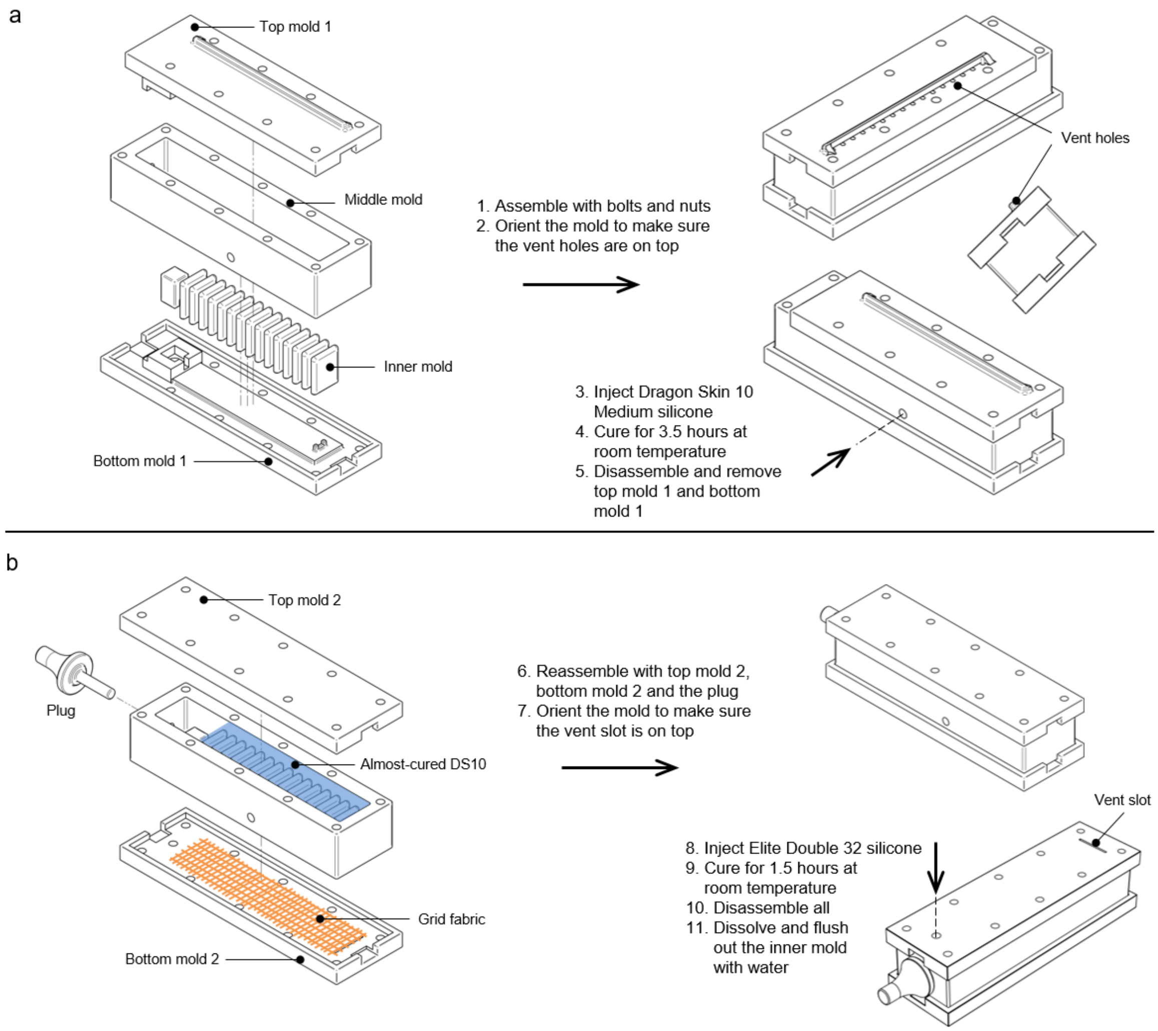

Figure S22: **Fabrication of the PneuNet actuator. a,** Molding the extensible layer with Dragon Skin 10 Medium silicone. **b,** Molding the inextensible layer with Elite Double 32 silicone. The inner mold was printed with BVOH filament on a Fused Filament Fabrication 3D printer (Ultimaker 3) and the other molds were printed with VeroClear on a PolyJet 3D printer (Eden260VS, Stratasys). After the curing and disassembly, the inner mold was flushed for 24 hours using a water pump in parallel with a tunable flow resistor for venting.

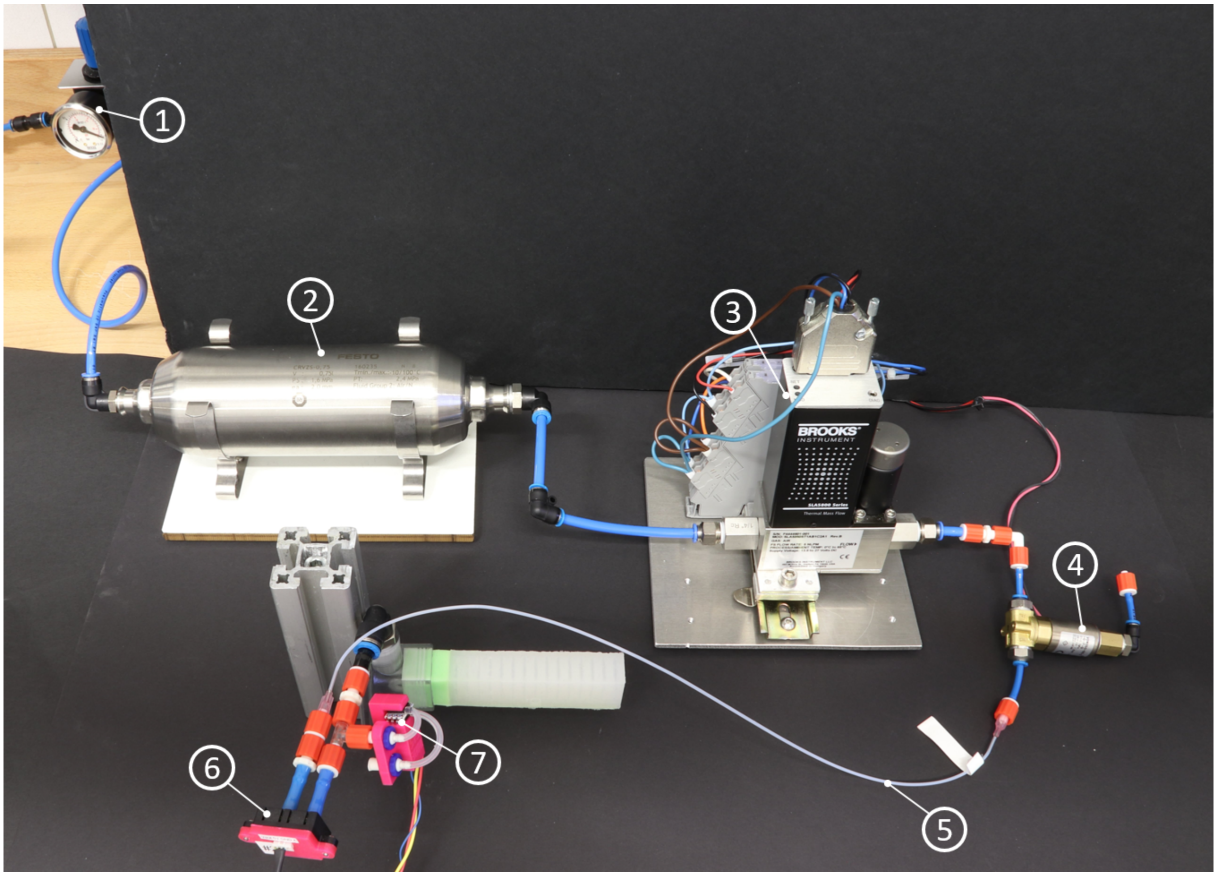

Figure S23: **Experimental setup for flow control with pressure measurement.** 1) Wall-mounted pressure regulator. 2) 0.75 L air tank. 3) Mass flow controller. 4) Solenoid valve. 5) Flow resistor. 6) Bidirectional flow sensor. 7) Pressure sensor.

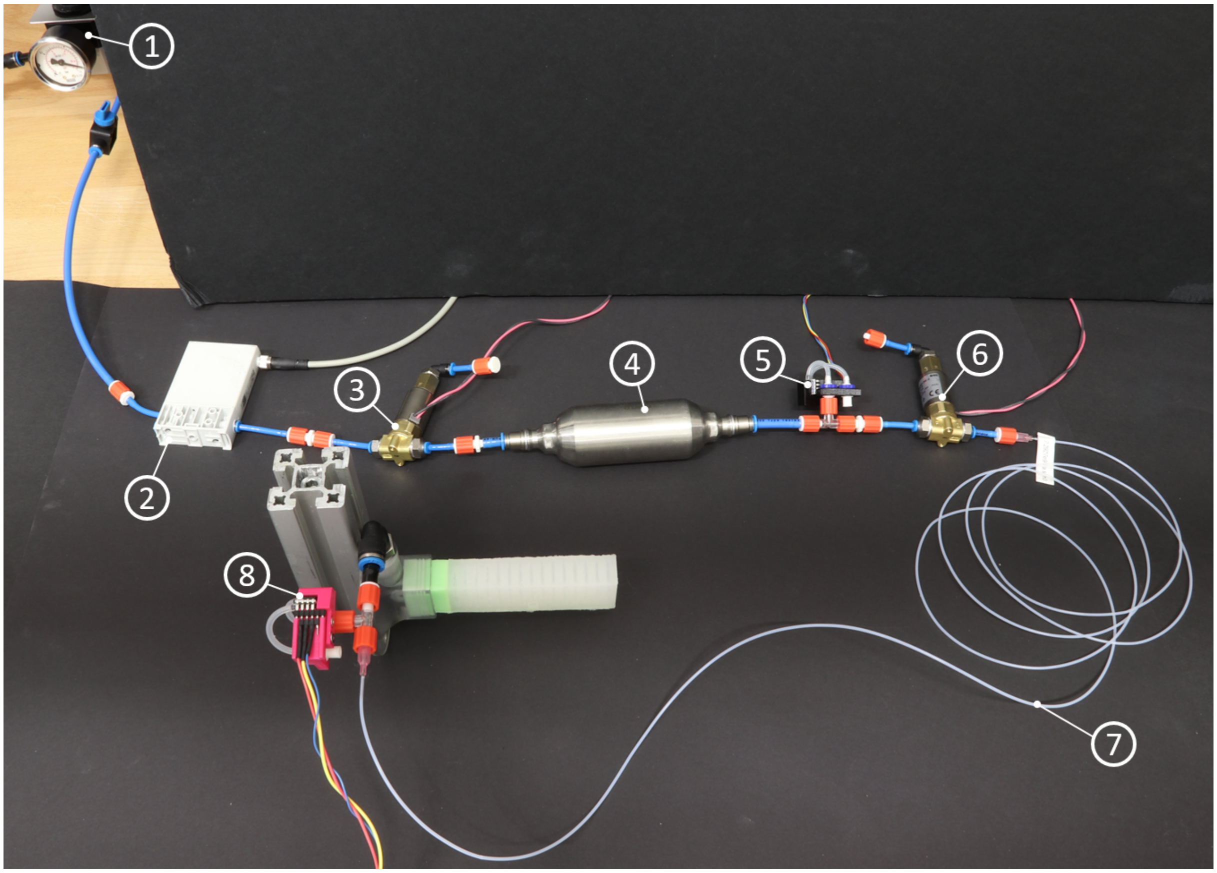

Figure S24: **Experimental setup for pressure control with pressure measurement.** 1) Wall-mounted pressure regulator. 2) Proportional pressure regulator. 3) Solenoid valve. 4) 0.1 L air tank. 5) Pressure sensor. 6) Solenoid valve. 7) Flow resistor. 8) Pressure sensor.

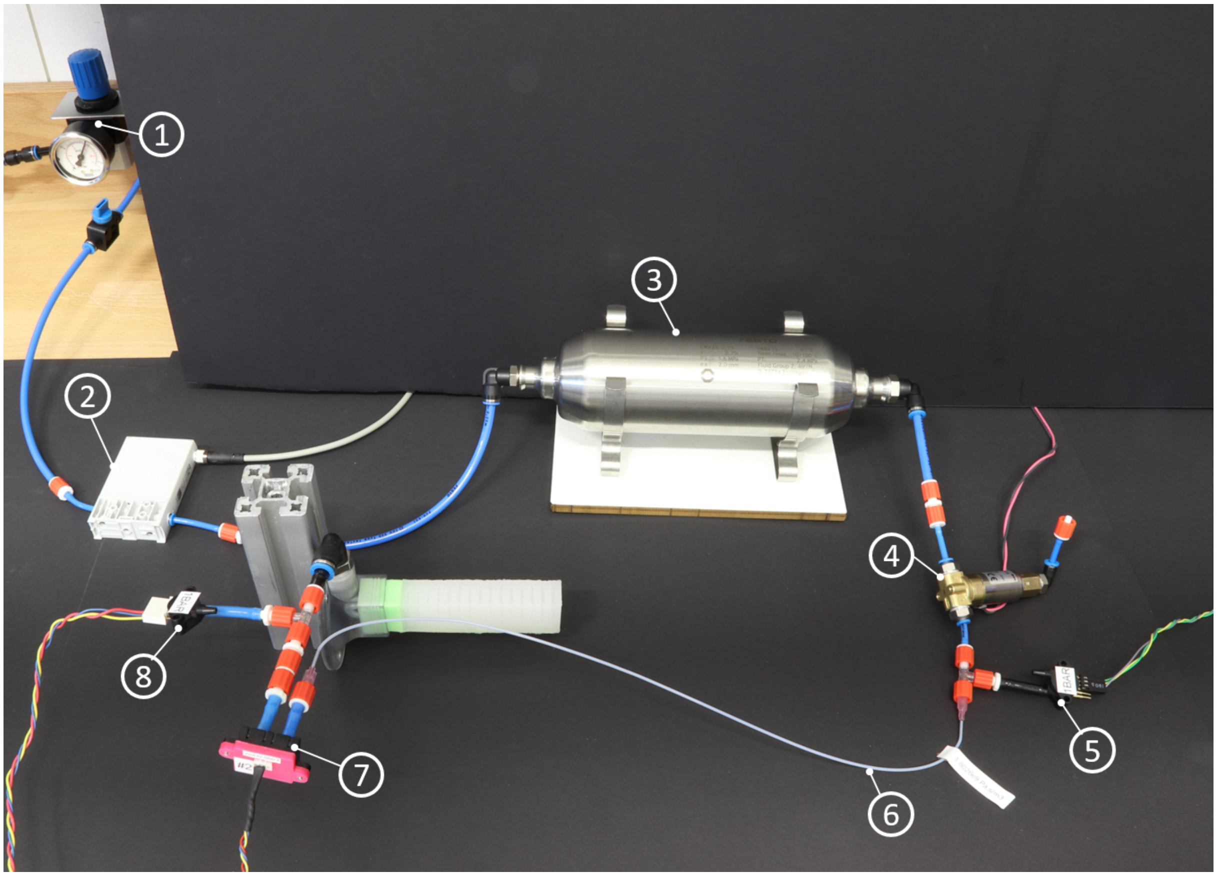

Figure S25: **Experimental setup for pressure control with flow measurement.** 1) Wall-mounted pressure regulator. 2) Proportional pressure regulator. 3) 0.75 L air tank. 4) Solenoid valve. 5) Pressure sensor. 6) Flow resistor. 7) Bidirectional flow sensor. 8) Pressure sensor.

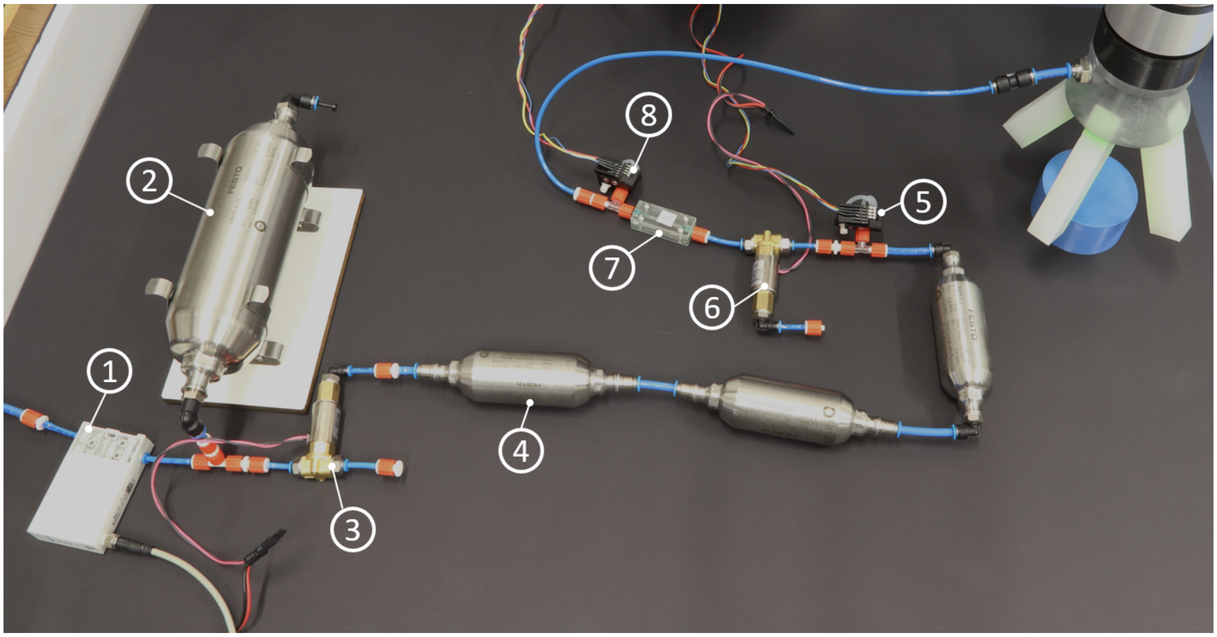

Figure S26: **Experimental setup for size sensing with soft gripper.** 1) Proportional pressure regulator. 2) 0.75 L air tank. 3) Solenoid valve. 4) Three 0.1 L air tank in series. 5) Pressure sensor. 6) Solenoid valve. 7) Flow resistor. 8) Pressure sensor.

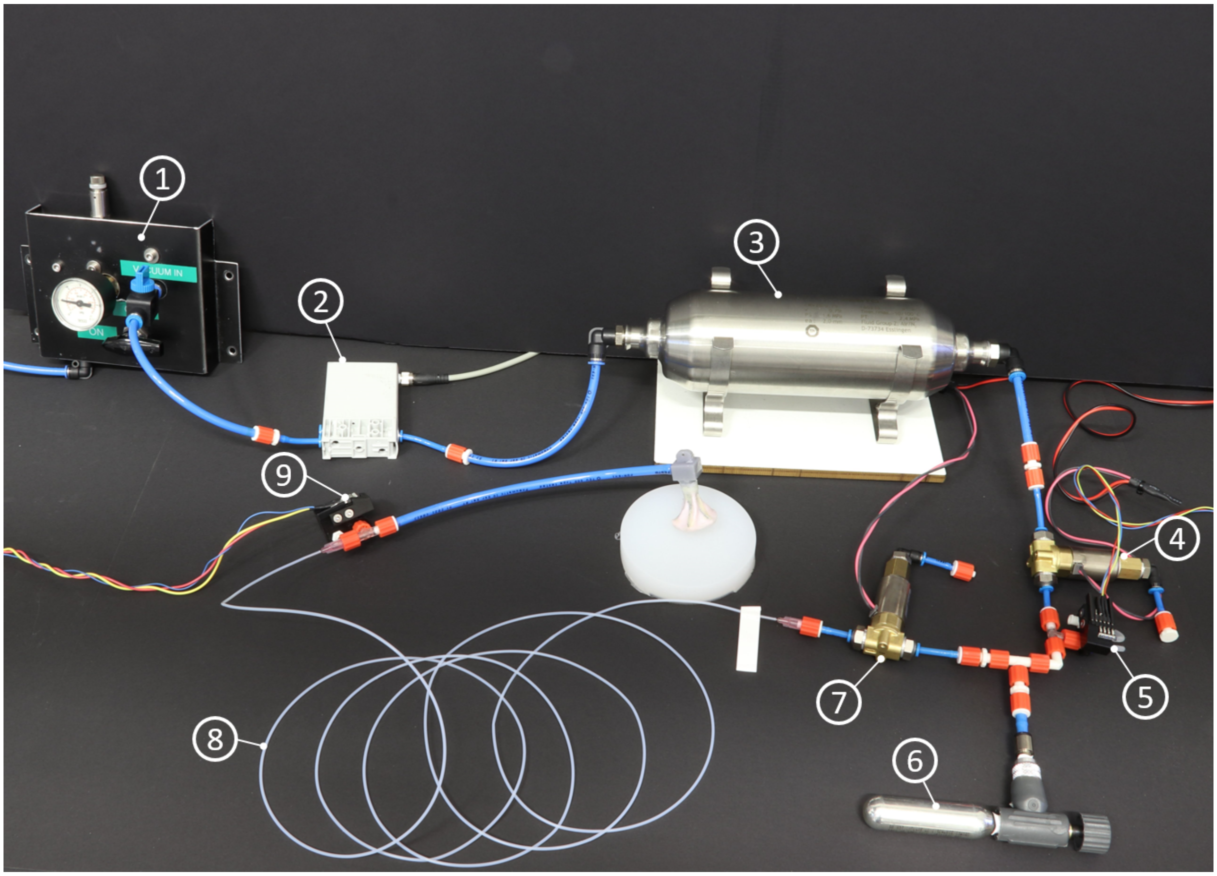

Figure S27: **Experimental setup for the surface stiffness sensing with the suction-based soft gripper.** 1) Vacuum pressure regulator. 2) Proportional pressure regulator. 3) 0.75 L air tank. 4) Solenoid valve. 5) Pressure sensor. 6) 15 ml air tank. 7) Solenoid valve. 8) Flow resistor. 9) Pressure sensor.

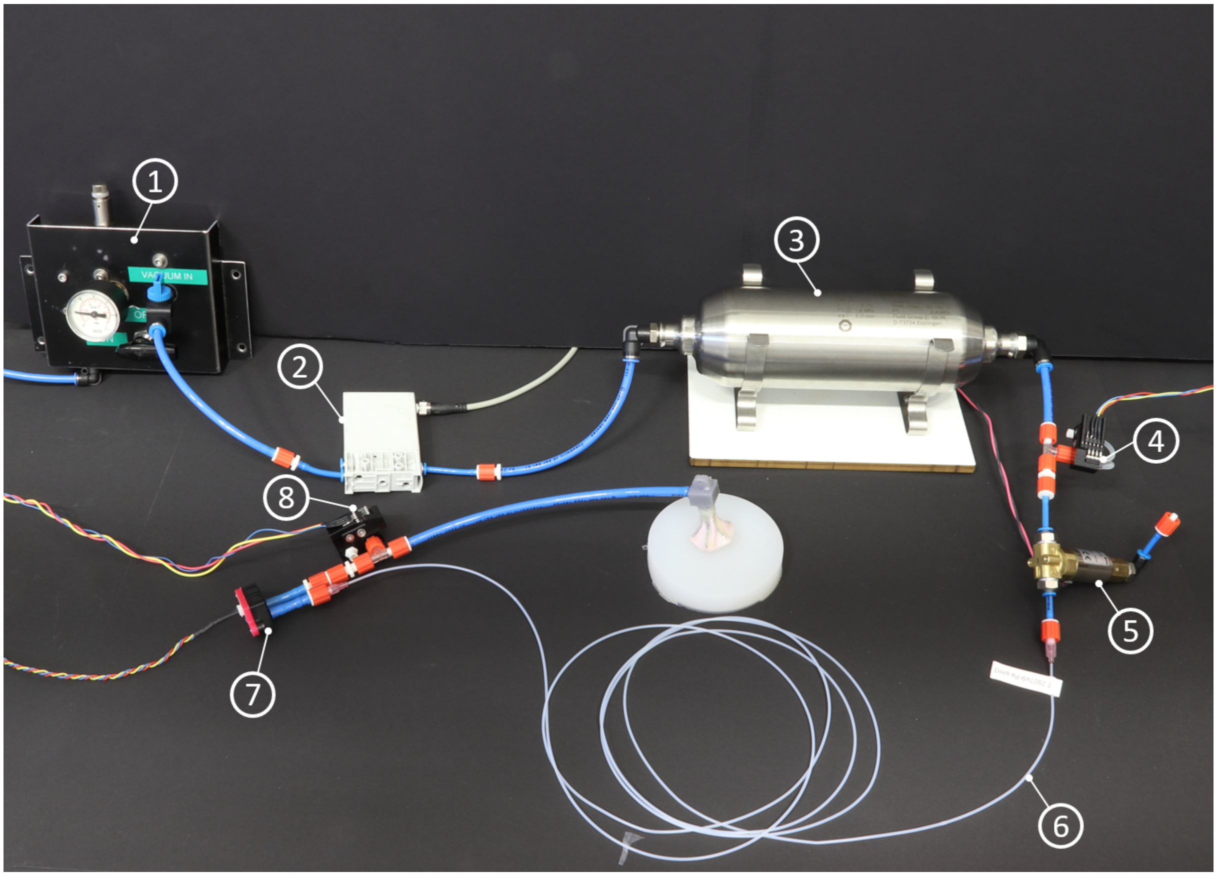

Figure S28: **Experimental setup for the characterization of pressure-volume response of the suction-based soft gripper.** 1) Vacuum pressure regulator. 2) Proportional pressure regulator. 3) 0.75 L air tank. 4) Pressure sensor. 5) Solenoid valve. 6) Flow resistor. 7) Bidirectional flow sensor. 8) Pressure sensor.

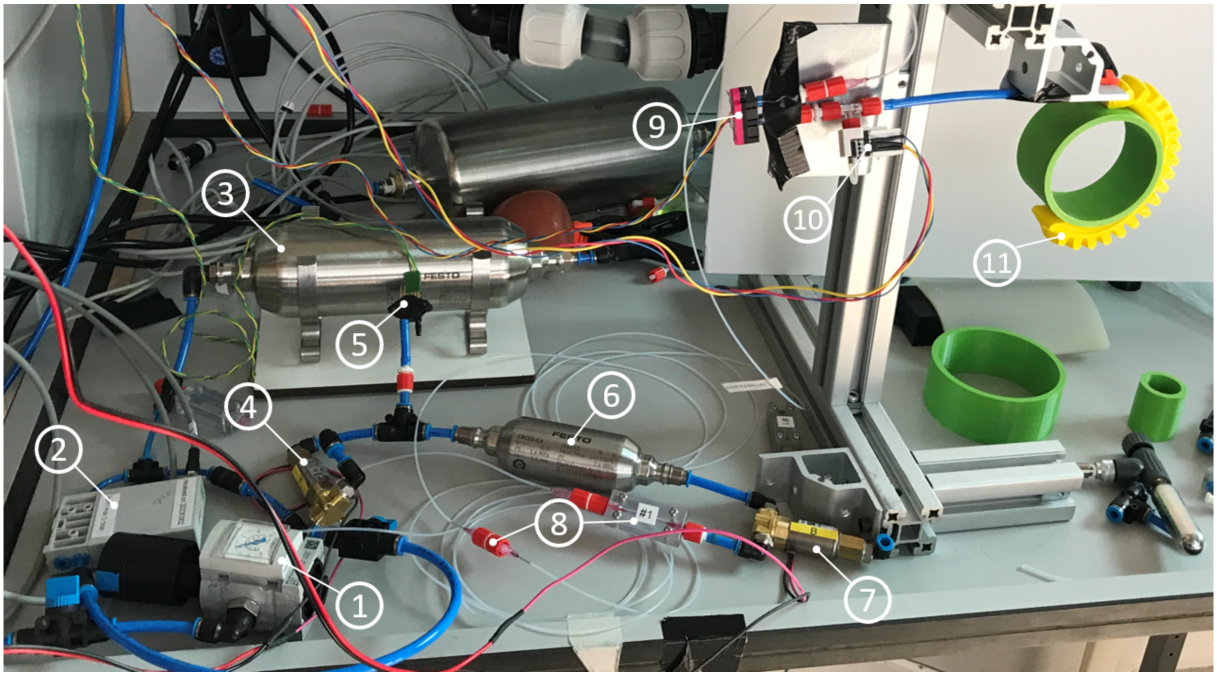

Figure S29: **Experimental setup for the size sensing with TPU bending actuator.** 1) Wall-mounted pressure regulator. 2) Proportional pressure regulator. 3) 0.75 L air tank. 4) Solenoid valve (VDW250-5G-1-01F-Q, SMC). 5) Pressure sensor. 6) 0.1 L air tank. 7) Solenoid valve (VDW250-5G-1-01F-Q, SMC). 8) Flow resistor. 9) Bidirectional flow sensor. 10) Pressure sensor. 11) TPU bending actuator.

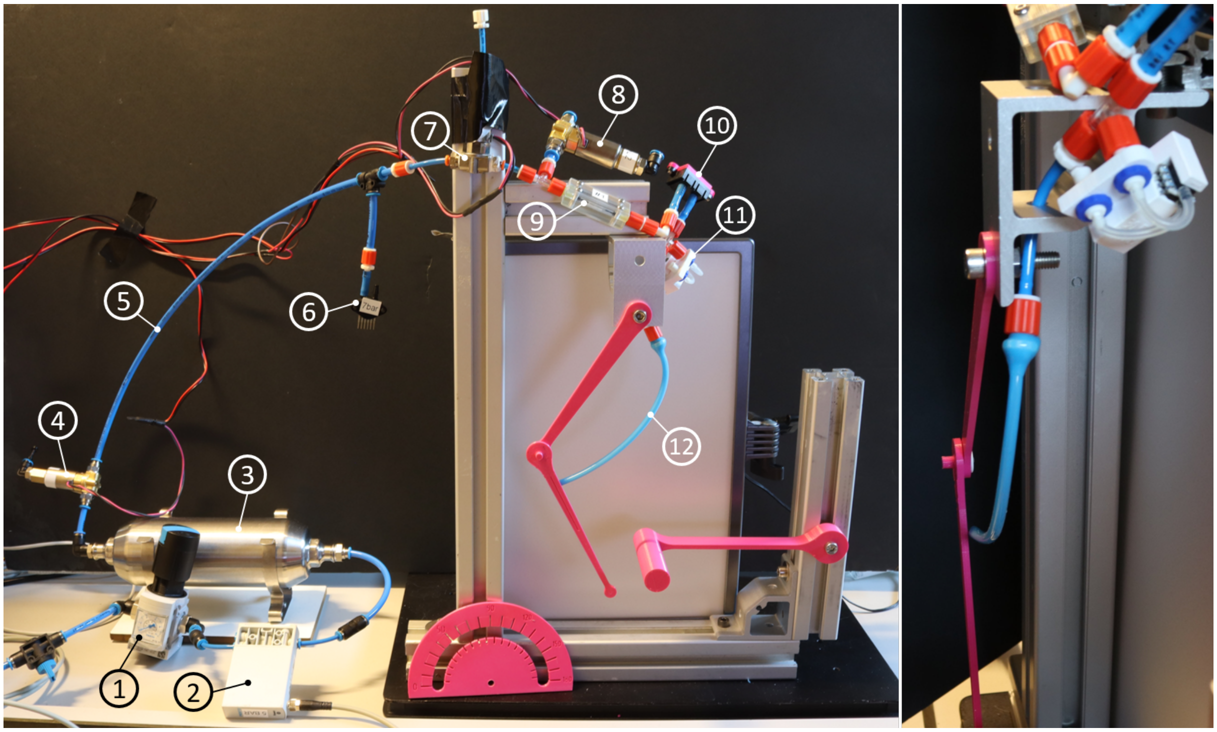

Figure S30: **Experimental setup for the angular displacement sensing with filament actuator.** 1) Wall-mounted pressure regulator. 2) Proportional pressure regulator. 3) 0.75 L air tank. 4) Solenoid valve. 5) Pressurized air pipe. 6) Pressure sensor. 7) Solenoid valve. 8) Solenoid valve. 9) Flow resistor. 10) Flow sensor. 11) Pressure sensor. 12) Filament actuator. Note that this setup contains more than the minimum number of required components for the sensing strategy to work. Solenoid valves 7) and 8) were arranged in such way to avoid leakage through the valves at high actuation pressure required by the filament actuator. Flow sensor 10) was used to obtain the full pressure-volume responses of both the pressurized air pipe and filament actuator and the flow resistor 9) was used to restrict the flow within the range of the flow sensor.

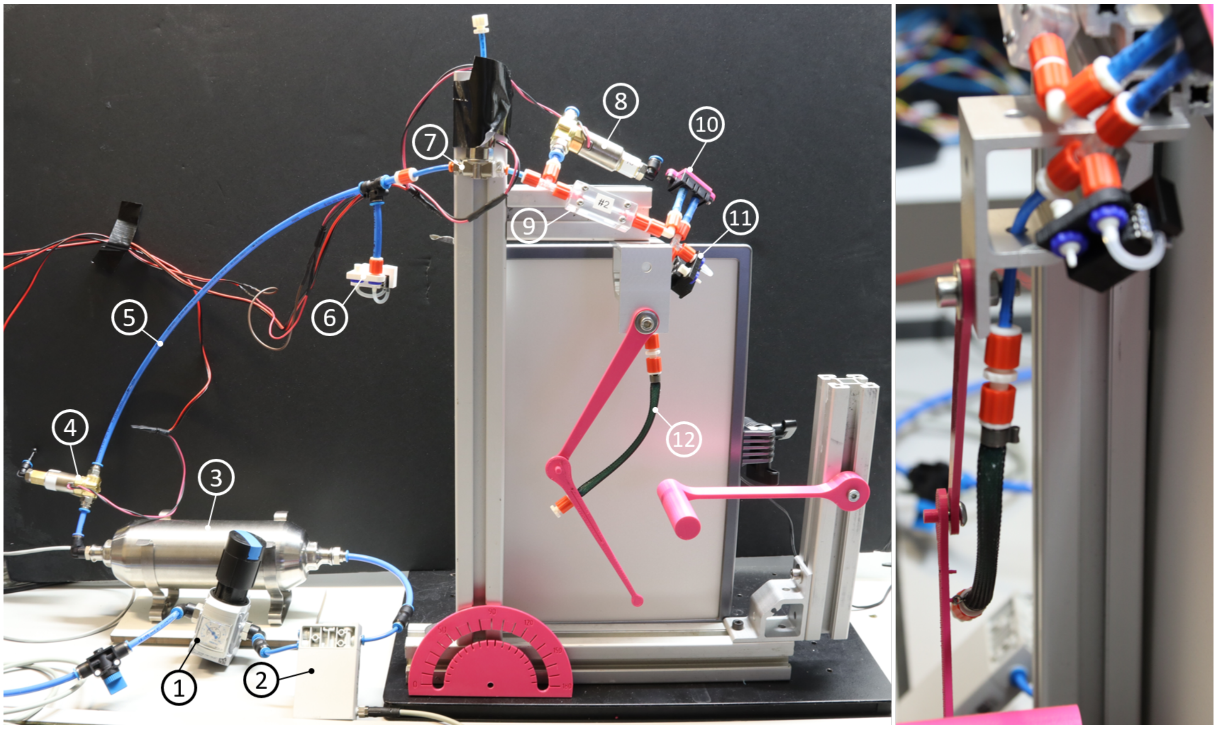

Figure S31: **Experimental setup for the angular displacement sensing with McKibben actuator.** 1) Wall-mounted pressure regulator. 2) Proportional pressure regulator. 3) 0.75 L air tank. 4) Solenoid valve. 5) Pressurized air pipe. 6) Pressure sensor. 7) Solenoid valve. 8) Solenoid valve. 9) Flow resistor. 10) Flow sensor. 11) Pressure sensor. 12) McKibben actuator. The setup was kept the same as that for the filament actuator in Fig. S30 except for the pressure sensors 6) and 11) which were selected to suit the pressure range of the McKibben actuator.

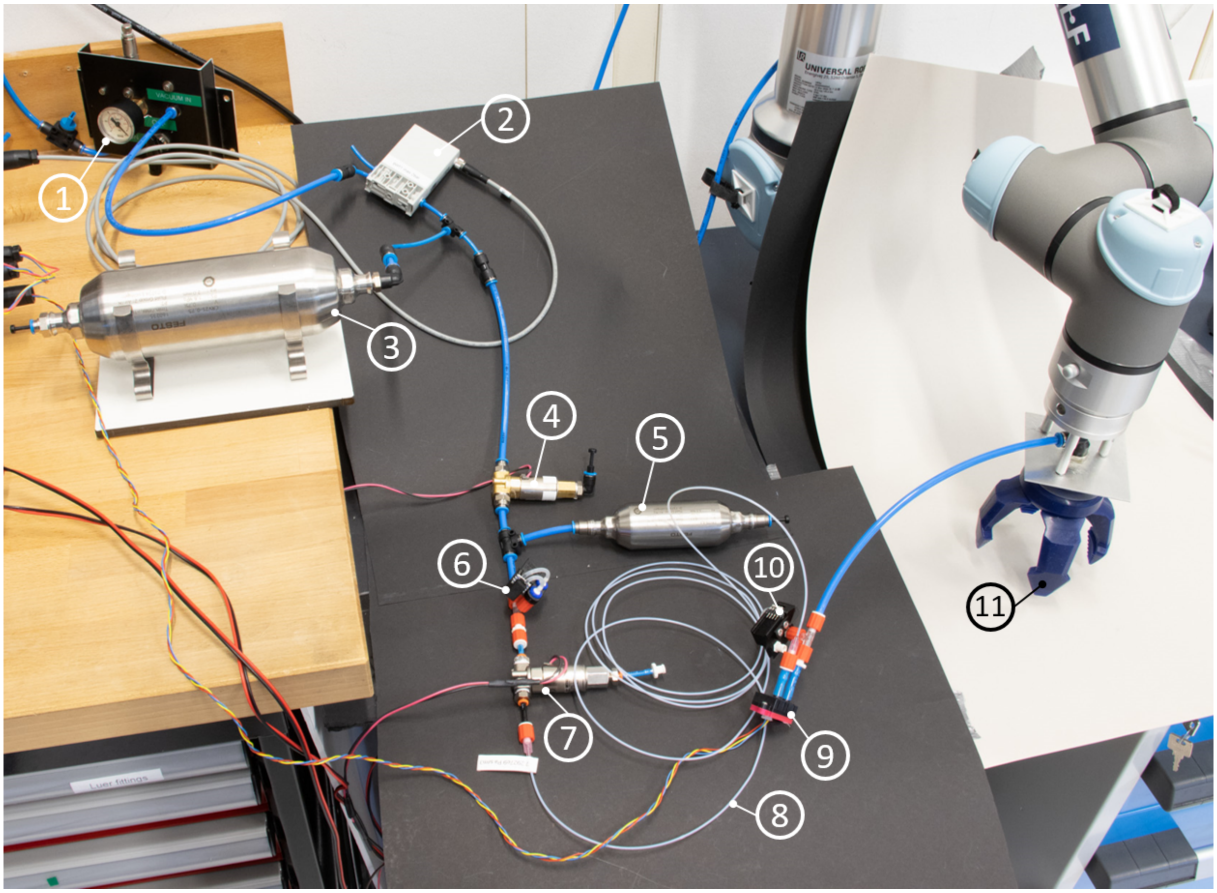

Figure S32: **Experimental setup for the size sensing with vacuum-powered commercial soft gripper.** 1) Vacuum pressure regulator. 2) Proportional pressure regulator. 3) 0.75 L air tank. 4) Solenoid valve. 5) 0.1 L air tank. 6) Pressure sensor. 7) Solenoid valve. 8) Flow resistor. 9) Flow sensor. 10) Pressure sensor. 11) Commercial soft gripper. Note that the flow sensor 9) was used to obtain the full pressure-volume responses of both the air tank and the soft gripper and the flow resistor 8) was used to restrict the flow within the range of the flow sensor.

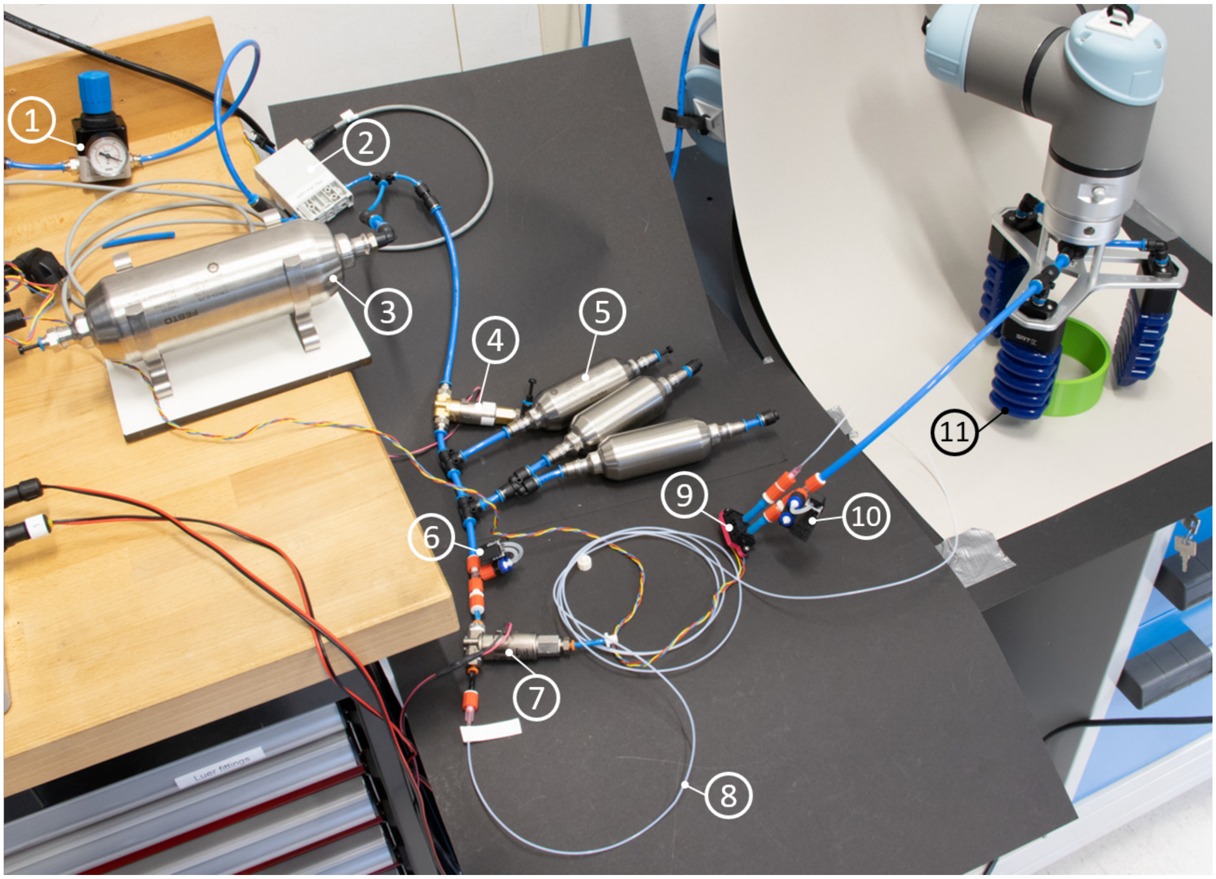

Figure S33: **Experimental setup for the size sensing with commercial soft PneuNet gripper.** 1) Wall-mounted pressure regulator. 2) Proportional pressure regulator. 3) 0.75 L air tank. 4) Solenoid valve. 5) Three 0.1 L air tanks. 6) Pressure sensor. 7) Solenoid valve. 8) Flow resistor. 9) Flow sensor. 10) Pressure sensor. 11) Commercial soft PneuNet gripper. Note that the flow sensor 9) was used to obtain the full pressure-volume responses of both the air tank and the soft gripper and the flow resistor 8) was used to restrict the flow within the range of the flow sensor.

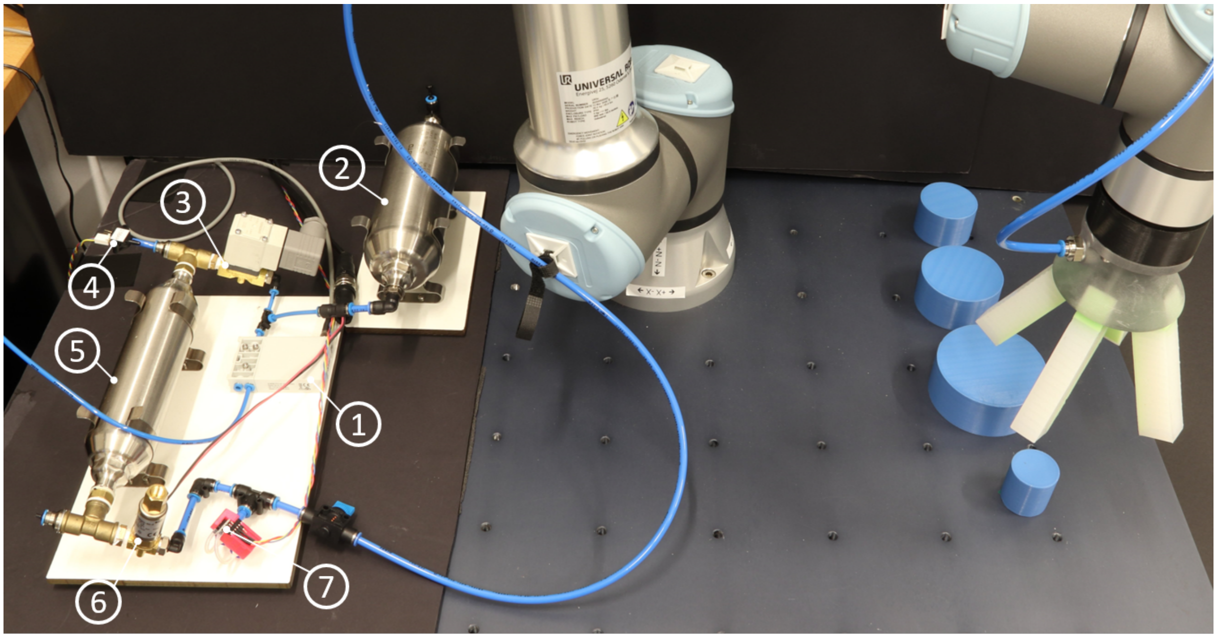

Figure S34: **Experimental setup for the sorting experiment.** 1) Proportional pressure regulator. 2) 0.75 L air tank. 3) Solenoid valve. 4) Pressure sensor. 5) 0.4 L air tank. 6) Solenoid valve. 7) Pressure sensor.

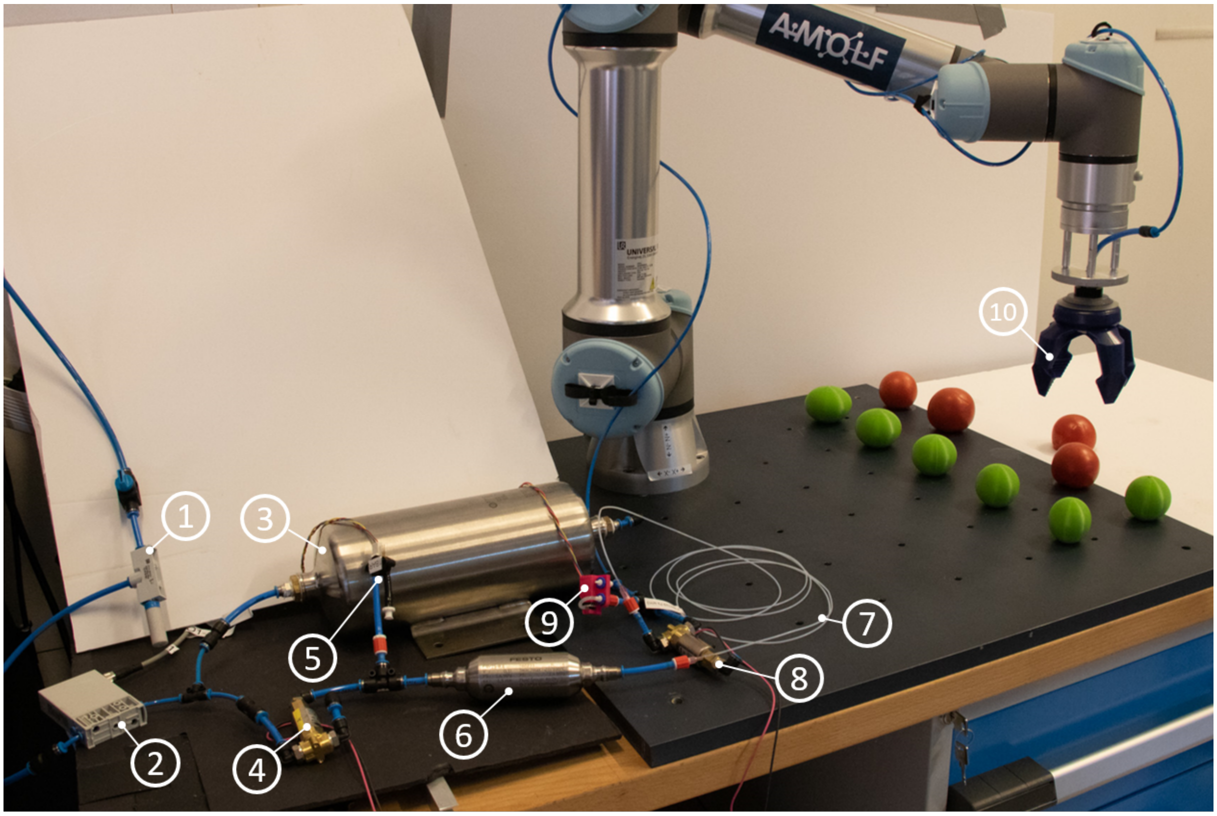

Figure S35: **Experimental setup for the tomato ripeness sensing experiment.** 1) Vacuum generator (FVN-05-H-T3-PQ2-VQ2-RO1, Festo) connected to a 7 bar compressed air source. 2) Proportional pressure regulator. 3) 2 L air tank. 4) Solenoid valve (VDW250-5G-1-01F-Q, SMC). 5) Pressure sensor. 6) 0.1 L air tank. 7) flow resistor. 8) Solenoid valve (VDW250-5G-1-01F-Q, SMC). 9) Pressure sensor. 10) Commercial soft gripper.

References

[1] H.K. Yap, H.Y. Ng, C.-H. Yeow, High-force soft printable pneumatics for soft robotic applications, *Soft Robotics* **3**, 144–158 (2016).

[2] K.P. Becker, Y. Chen, R.J. Wood, Mechanically programmable dip molding of high aspect ratio soft actuator arrays, *Advanced Functional Materials* **30**, 1908919 (2020).

[3] A. Iniguez-Rabago, Y. Li, J.T. Overvelde, Exploring multistability in prismatic metamaterials through local actuation, *Nature Communications* **10**, 5577 (2019).
