# Supplementary material for: A retrofit sensing strategy for soft fluidic robots
Source: Nat Commun. 2024 Jan 15;15:539. doi: 10.1038/s41467-023-44517-z (PMC10789869; doi:10.1038/s41467-023-44517-z)
Supplement: Supplementary file 3 — Description of Additional Supplementary Files [file 41467_2023_44517_MOESM3_ESM.docx]

Supplementary videos for

**A Retrofit Sensing Strategy for Soft Fluidic Robots**

Shibo Zou^1^ , Sergio Picella^1,2^ , Jelle de Vries^1^ , Vera G. Kortman^3,4^ , Aimée Sakes^4^ , Johannes T. B. Overvelde^1,2^

^1^ Autonomous Matter Department, AMOLF, Amsterdam, 1098 XG, The Netherlands

^2^ Institute for Complex Molecular Systems and Department of Mechanical Engineering, Eindhoven University of Technology, Eindhoven, 5600 MB, The Netherlands

^3^ Department of Marine and Transport Technology, Delft University of Technology, Delft, 2628 CD, The Netherlands

^4^ Bio-Inspired Technology Group, Department of BioMechanical Engineering, Delft University of Technology, Delft, 2628 CD, The Netherlands

Corresponding author: Johannes T. B. Overvelde, overvelde@amolf.nl

File Name: Supplementary Video 1

Description: Fluidic sensing of the soft robot-environment interaction: 1. Fluidic response of a soft actuator inflated onto a rigid plate. A PneuNet actuator is inflated with standard air volume of 28.7 ml onto a rigid plate from different heights *h*. Due to the compliance of the soft body, the interaction with the plate influences the fluidic response of the soft actuator. This fluidic response can be used to infer the interaction. 2. Size sensing. A soft gripper is actuated by a 0.3 L air tank that is initially pressurized at 63 kPa. The final equilibrium pneumatic pressure of the gripper can be used to infer the size of the cylindrical object in the gripper.

File Name: Supplementary Video 2

Description: Time-enabled sensing versatility: 1. Shape sensing. A soft gripper is actuated by a 0.3 L air tank that is initially at a pressure of 63 kPa. The pneumatic pressure of the gripper is measured over time and compared to a reference response where the gripper closes without touching anything. Time of contacts can be determined from the pressure-time response and used to infer the aspect ratio of the rectangular object. 2. Stiffness sensing. A PneuNet actuator is actuated by a 0.1 L air tank that is initially at a pressure of 49.9 kPa. The pressure of the actuator is measured over time and compared to a reference response where the actuator is inflated without touching anything. The time of contact can be determined from the pressure-time response and, together with the final equilibrium pressure, can be used to compare the stiffness values of different objects. 3. Profile scanning. A PneuNet actuator is actuated by a 0.1 L air tank that is initially at a pressure of 49.9 kPa. The actuator is moved horizontally by a robotic arm for 100 mm at a speed of 2 mm/s. The pressure-time response of the actuator can be used to reconstruct the surface profile based on a calibration curve and a reference response where the actuator is inflated without touching the surface.

File Name: Supplementary Video 3

Description: Retrofitting the fluidic sensing approach: 1. Suction cup. A suction cup is actuated onto silicone samples with different shore moduli by a 15 ml air tank that is initially at a pressure of -62 kPa. The surface stiffness of the sample influences the pressure-volume response of the suction cup through the amount of reduced internal geometric volume of the suction cup, which can be used in return to infer the surface stiffness. 2. TPU bending actuator. A TPU bending actuator is actuated around cylindrical objects with different diameters by a 100 ml air tank that is initially at a pressure of 300 kPa. The size of the cylindrical object influences the pressure-volume response of the bending actuator. The equilibrium pneumatic pressure of the bending actuator can be used to infer the diameter of the cylindrical objects. 3. Filament actuator. A filament actuator is actuated by a 6.3 ml air pipe that is initially at a pressure of 265 kPa, and acts as a muscle to rotate an arm towards a stopper. The pressure-volume response of the filament actuator changes with the total angular displacement θ of the joint. The equilibrium pneumatic pressure of the filament actuator can be used to infer the angular displacement θ of the joint. 4. McKibben actuator. A McKibben actuator is actuated by a 6.3 ml air pipe that is initially at a pressure of 140 kPa, and acts as a muscle to rotate an arm towards a stopper. The pressure-volume response of the McKibben actuator changes with the total angular displacement θ of the joint. The equilibrium pneumatic pressure of the McKibben actuator can be used to infer the angular displacement θ of the joint. 5. Vacuum-powered commercial soft gripper. A vacuum-powered commercial soft gripper is actuated by a 0.1 L air tank that is initially pressurized at -60 kPa. The final equilibrium pneumatic pressure of the gripper can be used to infer the size of the cylindrical object in the gripper. 6. Commercial soft PneuNet gripper. A commercial soft PneuNet gripper is actuated by a 0.3 L air tank that is initially pressurized at 70 kPa. The final equilibrium pneumatic pressure of the gripper can be used to infer the size of the cylindrical object in the gripper.

File Name: Supplementary Video 4

Description: Closed-loop control with fluidic sensing: size sorting. Four cylindrical objects with different diameters in a random input order are sorted by a robotic arm based on a modified insertion sort algorithm. The gripper is actuated by a 0.4 L air tank that is initially pressurized at 64 kPa. The equilibrium pneumatic pressure of the gripper provides a fluidic sensing feedback that is used to compare the size of the gripped cylindrical objects without using any calibration curve. The fluidic sensing feedback also works for sorting random objects.

File Name: Supplementary Video 5

Description: Closed-loop control with fluidic sensing: tomato picking. Tomatoes with preprogrammed positions are picked and placed by the soft gripper. For each tomato, three attempts are made, in which the gripper is actuated by a 0.4 L air tank that is set to a pressure of 62 kPa, 70 kPa, 78 kPa, respectively. The equilibrium pneumatic pressure of the gripper after each attempt is compared with a corresponding reference response to determine whether the tomato is successfully picked or not.

File Name: Supplementary Video 6

Description: Closed-loop control with fluidic sensing: picking out the overripe tomato. One overripe tomato, three ripe tomatoes and one dummy are placed at preprogrammed positions. The vacuum-powered commercial soft gripper grasps the objects one by one to pick out the overripe tomato. The experiment includes one calibration cycle and five sensing cycles. The objects are shuffled randomly between sensing cycles. The size of the object *D*_0_ upon gripping can be inferred by either *t*_c_ or Δ*P*_max_. The final size of the object *D*_1_ after gripping can be inferred by Δ*P*_eq_. The ripeness of tomato can be evaluate with *D*_0_ - *D*_1_.
